# Hybrid Cluster Precursors of the LaZrO Insulator for Transistors: Properties of High-Temperature-Processed Films and Structures of Solutions, Gels, and Solids

**DOI:** 10.1038/srep29682

**Published:** 2016-07-14

**Authors:** Jinwang Li, Peixin Zhu, Daisuke Hirose, Shinji Kohara, Tatsuya Shimoda

**Affiliations:** 1 Green Device Research Center, Japan Advanced Institute of Science and Technology (JAIST), 2-13 Asahidai, Nomi, Ishikawa 923-1211, Japan; 2 Japan Science and Technology Agency (JST), ERATO, Shimoda Nano-Liquid Process Project, 2-13 Asahidai, Nomi, Ishikawa 923-1211, Japan; 3 Core Functionalities Development Center, Central Research Laboratories, DIC Corporation, 631 Sakado, Sakura, Chiba 285-8668, Japan; 4 School of Materials Science, Japan Advanced Institute of Science and Technology (JAIST), 1-1 Asahidai, Nomi, Ishikawa 923-1292, Japan; 5 SPring-8/Japan Synchrotron Radiation Research Institute (JASRI), 1-1-1 Kouto, Sayo-cho, Sayo-gun, Hyogo 679-5198, Japan; 6 Synchrotron X-Ray Group, Quantum Beam Unit, National Institute for Materials Science, 1-1-1 Kouto, Sayo-cho, Sayo-gun, Hyogo 679-5148, Japan

## Abstract

In the solution processing of oxide electronics, the structure of metal–organic precursors in solution and their effect on processability and on the final structure and properties of the oxide have rarely been studied. We have observed that hybrid clusters, having inorganic cores coordinated by organic ligands, are the typical form of metal–organic precursor structures. For insulating ternary LaZrO, improved synthesis of the cluster precursor under solvothermal conditions led to low-temperature deposition of the film at 200 °C, as we will report in another paper. In the current paper, we first briefly show that solvothermal synthesis of the precursor resulted in significantly improved insulating properties (e.g., two orders lower leakage current) of high-temperature-annealed films, and then focus on the structural analysis of the cluster precursors and annealed solids and relate the results to the significant improvement of properties by solvothermal treatment of solutions. A change in the cluster core toward structural unification was brought about by solvothermal treatment, resulting in higher uniformity and higher stability of clusters. The final structure of the material maintained the features of the core structure in solution, even after annealing at high temperatures. These results demonstrate the key role played by designing cluster structure in solution.

Solution processing and printing techniques have attracted significant attention because of their potential to solve the problems of low resource and energy efficiencies and high facility and processing costs in the conventional fabrication of electronic devices. Regarding the candidate materials, metal oxides are being employed in increasingly diverse applications, such as in display-driving devices, particularly transistors, because of their high electrical performance and thermal and chemical stability under ambient conditions. Solution processing of oxide transistors has been intensively studied in recent years, with several low-temperature (below 250 °C) approaches being developed and device fabrication on flexible substrates being demonstrated^1^,^2^,^3^,^4^,^5^,^6^,^7^,^8^,^9^.

Nevertheless, the application of solution-processed oxide transistors in practical devices remains challenging. First, the robustness of the process and the reliability and stability of the transistors need to be significantly improved for commercial applications. This requires a thorough understanding of the details of the structural evolution in the process and its sensitivity to processing parameters. For this purpose, the structure of the starting material, i.e., the solution, as well as its effect on the structural changes in the subsequent processing steps, has to be studied in detail. Unfortunately, although numerous studies on solution-processing of oxide transistors have been reported, detailed investigation into the precursor structure and its optimization is rare. Second, more materials should be developed by solution processing. The advances hitherto achieved have mainly involved semiconducting oxides. Solution-processing routes to excellent gate insulators having both low and high dielectric constants and superior interfacing properties, as well as highly conductive materials, are now needed.

We have been studying the solution processing and printing of oxide thin-film transistors (TFTs). For this purpose, we have developed various solution-processable materials^10^,^11^,^12^,^13^,^14^,^15^,^16^. As a result, we have realized wholly solution-processed, all-oxide TFTs^17^,^18^, and developed a direct printing method, namely nano-rheology printing, for sub-micron oxide patterns and devices^17^. In this research, we found that precursor solutions were typically composed of hybrid clusters, comprising inorganic cores surrounded by ligands, which have completely different structures from those of the raw reagents^15^,^17^. Deformation during printing, thermal decomposition and densification, and final material structure are all closely correlated to the precursor structure. Because the materials evolve via a hybrid cluster (solution) → cluster gel (dried) → solid route, the study and design of the precursor cluster are very important, as has been demonstrated in our published work on the solution processing of semiconducting indium oxide, conducting ruthenium oxide, and ruthenium metal^15^,^17^, as well as in our unpublished work on other oxides. The synthesis of a stable precursor cluster in a solution is the first step toward a robust process.

High-dielectric-constant (high-*k*) materials are in demand for low-voltage operation and further miniaturization of transistors. We have developed an insulating material, LaZrO, based on the study of precursor clusters. Both lanthanum oxide and zirconium oxide are typical high-*k* materials, having dielectric constant values in the range 20–25. However, lanthanum oxide is hygroscopic and both oxides are polycrystalline. An amorphous structure is preferred for a sharp insulator/channel interface and for eliminating leakage through crystal boundaries. The combination of these two oxides led to the insulating LaZrO, which is a stable amorphous oxide (refs 18, 19; also see Figure S1 for a transmission electron microscopy image and an electron diffraction pattern) with a dielectric constant in the range 20–25. LaZrO has shown some excellent properties in our all-oxide TFTs^17^,^18^,^19^, but leakage needs to be further suppressed and a processing temperature that is compatible with plastic substrates is highly desirable. In another paper^20^, we will show that the light absorption and decomposition properties of LaZrO precursor solutions were significantly improved by a solvothermal treatment, leading to low-temperature (200 °C) decomposition and densification under ultraviolet (UV) light for TFT fabrication. In the current paper, we analyze the short (nearest and next-nearest neighbors of atoms) and medium (a few nanometers) range structures of solutions, gels, and solids using synchrotron high-energy X-ray diffraction (HEXRD), X-ray absorption fine structure (XAFS) spectroscopy, and other methods, which suggest a close structural relationship between the clusters in the solutions and the final solids even after annealing at high temperatures. Hence, the solvothermal treatment of precursor solutions has fundamental effects on both films processed at low temperatures and those annealed at high temperatures.

## Experimental Methods

### Synthesis of Precursor Solutions

In a typical experiment, lanthanum(III) acetate 1.5-hydrate (0.686 g, 99.99%, Kanto Kagaku) and zirconium(IV) butoxide solution (0.959 g, 80 wt. % in 1-butanol, Aldrich) were each dissolved in appropriate amounts of propionic acid (>99.0%, Kanto Chemical) in capped glass vials with magnetic stirring for 30 min on a hotplate set at 110 °C to produce 0.2 mol/kg La and Zr solutions (10 g of each). The Zr compound was handled in a dry nitrogen-filled glovebox before being sealed in the vial. After cooling to room temperature, the two solutions were mixed to obtain LaZrO precursor solutions with La/Zr molar ratio of 3/7 or 1/1. For solvothermal treatment, 10 g of LaZrO precursor solution was sealed in an autoclave (AC) with a 50 ml PTFE inner container (HUT-50, San-Ai Kagaku Co. Ltd.) and heated at 160–180 °C on an autoclave heater (RDV-TMS-50, San-Ai Kagaku Co. Ltd.) for 2–5 h with magnetic stirring.

### Film Deposition

All solutions for film deposition were filtered through a 0.2-μm-pore filter. Pt/SiO_2_/Si substrates, typically 2 cm × 2 cm in size, were cleaned using oxygen plasma before deposition. The precursor LaZrO solution was spin-coated onto a substrate at 2000 rpm for 25 s, and then, dried at 250 °C for 5 min. The spin-coating and drying cycles were performed five times. Finally, the film was annealed at 400–600 °C in oxygen for 20 min in a rapid thermal annealing furnace.

For the measurement of insulating properties (current–voltage and dielectric–frequency relations), Pt top electrodes (100-nm-thick and 200–500 μm in diameter) were deposited through a metal mask using radio-frequency plasma sputtering at room temperature to form a capacitor structure. Post-annealing was performed at the same temperature as that of film deposition for 10 min.

### Thermal Gravimetric/Differential Thermal Analysis (TG/DTA)

The solutions were dried at 150 °C for 1 h in air to form gels. The thermal decomposition of these gels was studied using TG/DTA in air at a heating rate of 10 K/min (TG/DTA 6200, SII).

### Mass Spectrometry (MS)

Cryospray-ionization Fourier-transform ion-cyclotron-resonance mass spectrometry (CSI-FT-ICR-MS) of the precursor solutions was performed on a Solarix-JA system (Bruker Daltonics). The CSI method ionizes molecules without any damage by cooling them down to the temperature of liquid nitrogen. The solutions were diluted by a factor of 100 with methanol for the measurements. The online ChemCalc tool^21^ was used to calculate simulated spectra for assignment purposes.

### High-Energy X-Ray Diffraction (HEXRD)

HEXRD experiments using a synchrotron radiation source were performed for solutions, gels, and annealed powders at the beamline BL04B2 of the SPring-8 facility, Japan. The gels were prepared by drying the solutions in air at 150 °C for 1 h, and the powders were synthesized by annealing the gels at 500 °C for 1 h in air. The samples were loaded into 2-mm-diameter quartz capillaries and measured in air. The energy of the X-rays was 61.4 keV and the measurement was conducted by the 2*θ* scan method over the angular range 0.3°–48° (corresponding to *Q* = 0.2–25 Å^−1^). The total structure factor, *S*(*Q*), was calculated from the dependence of the measured intensity on 2*θ* (angle). The pair distribution function, *G*(*r*), and the total correlation function, *T*(*r*), were calculated by Fourier transformation of *S*(*Q*).

### X-Ray Absorption Fine Structure (XAFS) Analysis

XAFS measurements of solutions, gels, and annealed powders were conducted at the beamline BL14B2 of the SPring-8 facility for the La-K and Zr-K edges. The transmittance mode was used. The program package Demeter^22^ including ATHENA and ARTEMIS, was used for data processing and structural fitting. A structural model based on the Zr6 cluster^23^ (also see Figure S2(a)) was used for solutions and gels and a cubic ZrO_2_ model^24^ (also see Figure S2(b)) was employed for the annealed powders. This selection of models was based on previous studies of the Zr-carboxylate system^23^ and our MS and HEXRD data, which are presented below. The Zr–O bonds in solutions and gels were classified into two distances. A monoclinic ZrO_2_ powder sample was used as a reference, from which the amplitude reduction factor for Zr was calculated.

### Other Analyses

The film thicknesses were evaluated through ellipsometry (Sopra GES-5E Ellipsometer, SEMILAB Japan K.K.). Film densities were measured using the X-ray reflectivity method (X’Pert PRO MRD, PANalytical). The surface morphologies were investigated through atomic force microscopy (AFM; NanoNavi, SII). Elemental compositions of the thin films were obtained from combinational Rutherford backscattering spectrometry, nuclear reaction analysis, and hydrogen forward scattering spectrometry on Pelletron 3SDH (National Electrostatics Corp.) at Toray Research Center, Inc., Japan, with estimated precisions of ±0.5 atom% for C and H, ±1 atom% for La and Zr, and ±4 atom% for O. High-resolution transmission electron microscopy was performed by the Kobelco Research Institute Inc. Impedance measurements of the Pt/LaZrO/Pt capacitor samples were performed using an SI 1260 Impedance/Gain-Phase Analyzer (Solartron Analytical). The current–voltage characteristics of these capacitor samples were measured using a 4155C Semiconductor Parameter Analyzer (Agilent).

## Results and Discussion

Hereafter, we use LZ37 and LZ55 to refer to samples with La/Zr atomic ratios of 3/7 and 5/5, respectively, and AC160-*x*h and AC180-*x*h to indicate solvothermal treatment at 160 °C and 180 °C, respectively, for *x* h, where *x* is a number.

### Properties of Films Prepared at High Temperatures (400–600 °C)

**Figure 1:**
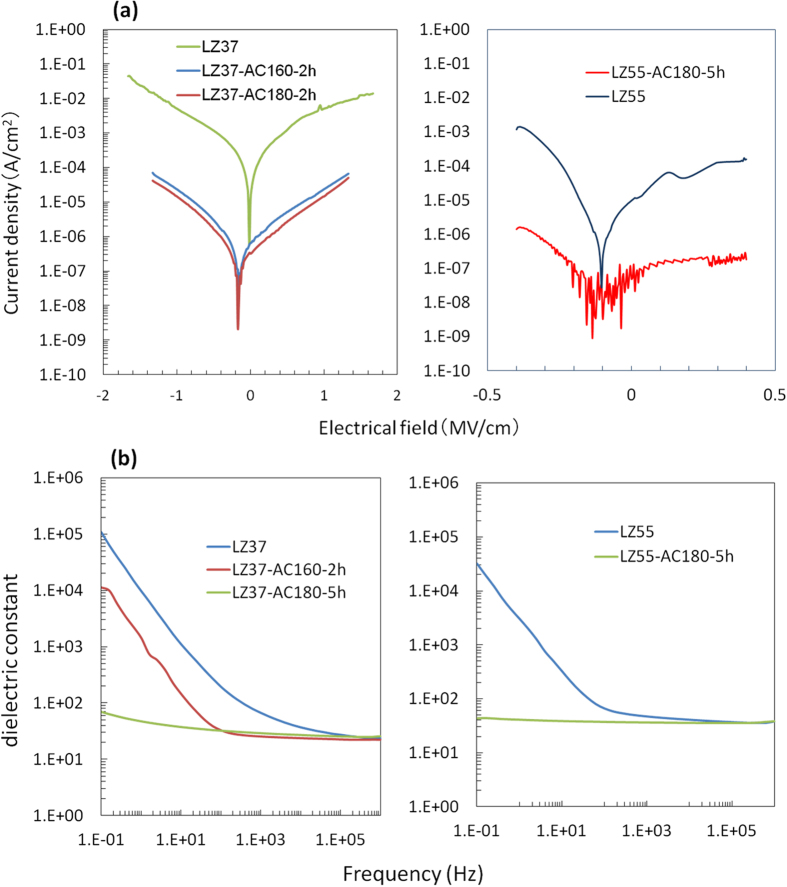
(**a**) Current density versus electric field and (**b**) dielectric constant versus frequency for LZ37 films annealed at 400 °C (left panels) and for LZ55 films annealed at 600 °C (right panels).

Before the presentation of the structural analyses, we describe the effect of solvothermal treatment on the properties of films annealed at high temperatures. Figure 1 shows the electrical properties of the annealed LaZrO films. We first measured the dependence of leakage current on electric field and found that the leakage current decreased by two orders after solvothermal treatment of the precursor solution (Fig. 1(a)). We then performed impedance measurements to evaluate the frequency dependence of the dielectric properties (Fig. 1(b)). For a high-quality insulator material, the dielectric constant is stable down to low frequency. In the literature, such measurements have often been performed down to only 10 or 100 Hz. We conducted the measurements down to 0.1 Hz since we found that below 10 Hz, the apparent dielectric constant could increase significantly with decreasing frequency. Such low-frequency conditions are close to those in TFT measurements using DC scans. After solvothermal treatment, the frequency dependence of the dielectric constant significantly decreased, concurring with the decrease in leakage current, which suggests the same structural origin of the high frequency dependence of the dielectric constant and the large leakage in this material. Without the solvothermal treatment of the precursor solution, the apparent dielectric constant increased by as much as a few orders upon decreasing the frequency from 1 MHz to 0.1 Hz. In the literature, this phenomenon has been attributed to Maxwell–Wagner-type contributions of depletion layers at the interface between the sample and electrodes or at grain boundaries^25^. Because our sample-electrode contacts are prepared under the same conditions, the observed high apparent dielectric constant indicates non-uniformity or defects in the material, although the overall structure was found to be amorphous by regular laboratory XRD. The non-uniform structure is likely to have low-resistance leakage paths similar to those formed at the grain boundaries in a polycrystalline material. In contrast, after solvothermal treatment of the precursor solution, the measured dielectric constant varied only slightly or became almost constant in the frequency range from 1 MHz to 0.1 Hz, suggesting that a uniform material structure was formed. An ideal insulating material should have a stable dielectric constant over at least such a frequency range.

The densities of the films are presented in Table 1, together with the values relative to the density of a LaZrO single crystal (assumed to be 6.1 g cm^−3^ based on the values of La_2_Zr_2_O_7_, 6.06 g cm^−3^, and La_0.1_Zr_0.9_O_1.95_, 6.1–6.3 g cm^−3^)^26^,^27^. At the same annealing temperature, the density increased with solvothermal treatment of the precursor solution. (Higher annealing temperatures also increased the density, as is commonly observed). This indicates that solvothermal treatment led to a structure in solution that favored the formation of dense films.

### TG/DTA Analysis

**Figure 2:**
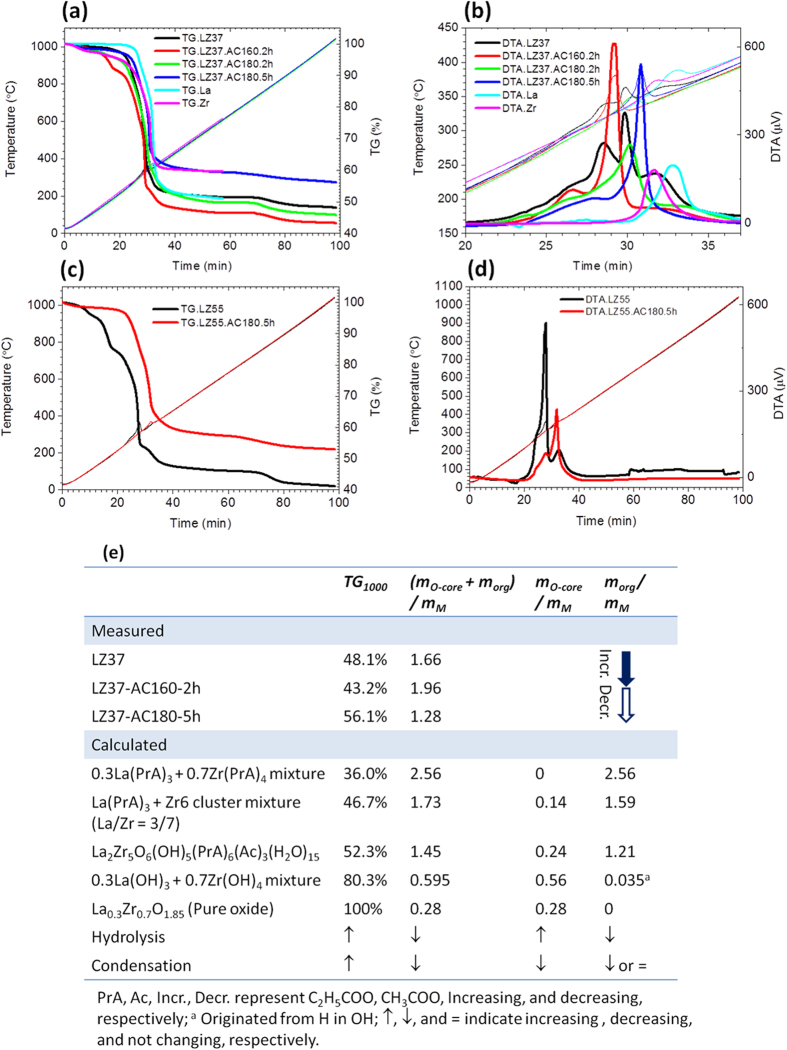
TG (**a**,**c**) and DTA (**b**,**d**) data for the precursor gels of the LZ37 (**a**,**b**) and LZ55 (**c**,**d**) samples. Panel (**e**) shows a detailed analysis of the TG data in (**a**) (see the main text for the explanation).

The TG/DTA results for the precursor gels obtained by drying solutions at 150 °C are shown in Fig. 2. In the TG data, the major weight loss located at 270–360 °C, which is accompanied by exothermic peaks in the DTA curves, was caused by oxidative decomposition of organic components, and the minor weight loss above 700 °C was attributed to further oxidation of the residual carbon. The intense heat release in the oxidative decomposition reaction caused an increase in the sample temperature that formed peaks of up to 80 °C in height on the linear temperature–time curves. Because of this non-monotonic temperature variation against time, the TG and DTA curves are plotted against time rather than against temperature in Fig. 2. The corresponding sample temperatures against time are also presented in the figure. For a plot of the TG and DTA data against sample temperature, see Figure S3. The temperatures corresponding to the exothermic DTA peaks are not the measured peak temperatures. Below, we will use an estimated average of the temperatures at the start and end points of each DTA peak as its corresponding temperature.

Three features were observed in the TG/DTA data for different La/Zr ratios, as shown in Fig. 2. Here, we use the specific data of the LZ37 samples to explain these features. First, the decomposition temperature of the organic components was affected by solvothermal treatment, as clearly shown in the DTA curves. The sample without solvothermal treatment showed several exothermic decomposition peaks in a broad range of 240–380 °C, with the two main peaks at around 327 °C and 345 °C. After solvothermal treatment at 160 °C for 2 h, only a main peak at ~325 °C and a minor one at ~296 °C were observed. Further increasing the temperature and time of the solvothermal treatment resulted in shifts of the decomposition peaks to higher temperatures; the dominating peaks shifted to 330 °C and 340 °C for solvothermal conditions of 180 °C/2 h and 180 °C/5 h, respectively. Such variation in the decomposition temperature indicates that solvothermal treatment led to structural unification, i.e., evolution from multiple, different cluster structures toward a single cluster structure, leading to a more uniform and stable cluster precursor. Second, both the La-only and Zr-only precursors showed decomposition temperatures higher than those of La-Zr precursors, indicating that the La and Zr components were not simply mechanically mixed, even before solvothermal treatment. Third, the residual masses (masses of the obtained oxide) after TG analysis were quite different for solvothermally treated precursor solutions; on moving from AC160 °C/2 h to AC180 °C/5 h via AC180 °C/2 h, the residual mass first decreased (AC160 °C/2 h) and then increased (AC180 °C/2 h), and finally, substantially exceeded (by 8%, AC180 °C/5 h) that of the sample obtained from an untreated precursor solution. This reflects the variation in the inorganic and organic contents in the precursors.

The third feature was further analyzed. The precursors were composed of hybrid clusters, as will be revealed in more detail below. We divide the compositions of the dried precursor into three types: (1) metal atoms (La and Zr), (2) oxygen atoms only bonded to the metal (O in M–O–M) and those bonded to both the metal and the hydrogen (O in M–O–H), and (3) other components (mainly organic ligands, including oxygen in RCOO bonded to a metal, where R = CH_3_ or CH_3_CH_2_), with their masses assigned to (1) *m*_*M*_, (2) *m*_*O-core*_, and (3) *m*_*org*_, respectively. The first two types of compositions constitute the inorganic core of the cluster. We assume that the composition at 1000 °C is a pure oxide in which the masses of the metals and oxygen are *αm*_*M*_ and *γαm*_*M*_, respectively, where 0 < *α* < 1 because of the partial evaporation of the metals during heating and *γ* is the mass ratio of oxygen to metal in the pure oxide. It follows that the relative residual mass at 1000 °C, *TG*_1000_, is


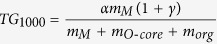


which can be transformed to


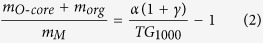


Because La and Zr are not volatile metals, *α *≈ 1. *γ* is a constant for a specific La/Zr ratio (*γ* = 0.28 for an oxide with La/Zr of 3/7, i.e., La_0.3_Zr_0.7_O_1.85_. See Supplementary Information). Accordingly, the values of (*m*_*O-core*_ + *m*_*org*_)/*m*_*M*_ were obtained from the experimental *TG*_1000_, and are listed in Fig. 2(e). Corresponding to the change in *TG*_1000_ values from 48.1% to 43.2% to 56.1%, the values of (*m*_*O-core*_ + *m*_*org*_)/*m*_*M*_ varied from 1.66 to 1.96 to 1.28 when increasing solvothermal temperatures/times (from no treatment to 160 °C/2 h to 180 °C/5 h).

We now consider the implications of this variation. In order to assist in understanding this, calculated data for simple mixtures of 0.3La(PrA)_3_ + 0.7Zr(PrA)_4_, of the Zr6 cluster [Zr_6_O_4_(OH)_4_(PrA)_12_] (ref. 23) and the La(PrA)_3_ molecule (PrA = propionate ligand CH_3_CH_2_COO), and for a possible LaZr cluster, La_2_Zr_5_O_6_(OH)_5_(PrA)_6_(Ac)_3_(H_2_O)_15_ (see analyses below; Ac = acetic ligand CH_3_COO), are presented in Fig. 2(e). These data indicate that the oxygen content of the core, *m*_*O-core*_/*m*_*M*_, increased, whereas the organic content, *m*_*org*_/*m*_*M*_, decreased with the development of cluster structures. This is because of the growth of the M–O–M network of the core, which is accompanied by the release of organic ligands. The corresponding (*m*_*O-core*_ + *m*_*org*_)/*m*_*M*_ values appear to be dominated by *m*_*org*_/*m*_*M*_ decreasing with the cluster development. However, the observed (*m*_*O-core*_ + *m*_*org*_)/*m*_*M*_ did not continuously decrease, but first increased from 1.66 to 1.96 before decreasing to 1.28.

To achieve further insight, we consider the possible reactions and their influence on the variation in *m*_*O-core*_/*m*_*M*_, *m*_*org*_/*m*_*M*_, and their total. We observed precipitation after solvothermal treatment at higher temperatures (e.g., 200 °C) and higher concentrations. This must be caused by the excessive development and growth of clusters into particles. The reactions are likely to be as follows. First, the organic ligand is replaced by a hydroxyl through a hydrolysis reaction in the presence of water


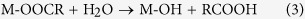


where M represents a metal atom at the cluster surface or in a metal–organic molecule. The water in the system was mainly generated in the esterification reaction between 1-butanol and propionic acid, which was found to occur quickly at room temperature according to detailed studies for the mixtures of zirconium alkoxides and carboxylic acids^23^,^28^. Second, M-OH and M-OOCR undergo condensation


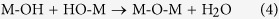



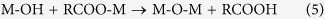


The hydrolysis leads to an increased *m*_*O-core*_/*m*_*M*_ (limited to a maximum of 0.56, or 2*γ*, see the calculated data for a mixture of La(OH)_3_ and Zr(OH)_4_ in Fig. 2(e)), and the condensation results in a decreased (reaction 4) or unchanged (reaction 5) *m*_*O-core*_/*m*_*M*_. Both hydrolysis and condensation (reaction 5) cause decreasing *m*_*org*_/*m*_*M*_ values. Because the mass of RCOO is around 4 times higher than that of OH, the (*m*_*O-core*_ + *m*_*org*_)/*m*_*M*_ value is dominated by *m*_*org*_/*m*_*M*_. As a result, the (*m*_*O-core*_ + *m*_*org*_)/*m*_*M*_ value decreases in all three reactions. These tendencies of *m*_*O-core*_/*m*_*M*_, *m*_*org*_/*m*_*M*_, and their total are also summarized in Fig. 2(e).

Accordingly, the observed increase in (*m*_*O-core*_ + *m*_*org*_)/*m*_*M*_ from 1.66 (without solvothermal treatment) to 1.96 (160 °C/2 h) cannot be a result of reactions (3)–(5). At least one of the reverse reactions must have occurred. This would allow decomposition of the initial Zr clusters and formation of new clusters incorporating La. This is supported by the above observation that the La and Zr compounds were not just mechanically mixed.

Therefore, the cluster development underwent two stages. The first stage is likely to be associated with the decomposition of the initial Zr clusters during their transition into La–Zr clusters, during which (*m*_*O-core*_ + *m*_*org*_)/*m*_*M*_ increased. The second stage involved further growth of the clusters, which finally led to a much lower (*m*_*O-core*_ + *m*_*org*_)/*m*_*M*_ value (1.28) than that before solvothermal treatment (1.66). The decrease in (*m*_*O-core*_ + *m*_*org*_)/*m*_*M*_ indicated less organic content in the precursor clusters.

In summary, the TG/DTA data suggest that the La compounds and Zr compounds were not simply mixed, but rather formed dominant structures containing both La and Zr that experienced structural unification to become more uniform and stable and contained less organics upon solvothermal treatment.

### MS Analysis

**Figure 3:**
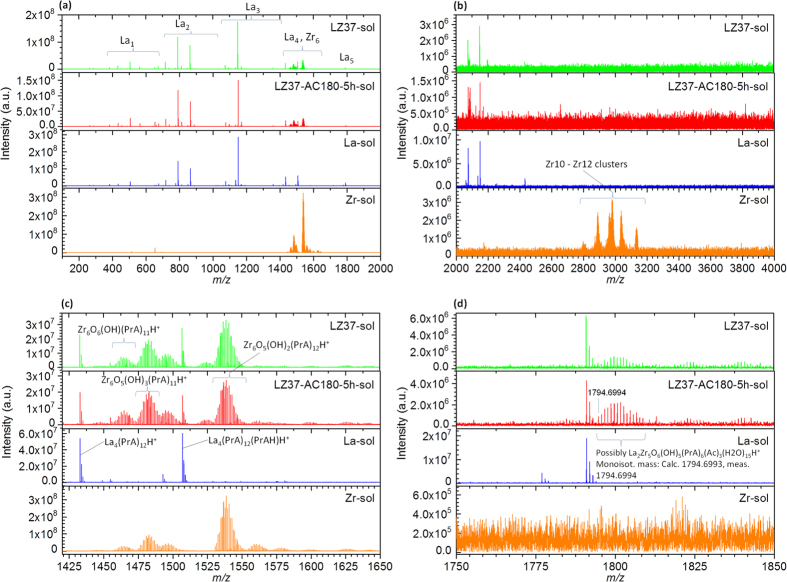
MS spectra in the *m*/*z* range (**a**) 0–2000 and (**b**) 2000–4000. Panel (**c**) is an expanded part of (**a**) in the *m*/*z* range 1420–1650, showing the Zr6 clusters. Panel (**d**) is an expanded part of (**a**) in the *m*/*z* range 1750–1850, showing the presence of clusters containing both La and Zr atoms in the LaZr solutions. For details of the assignments, see Figure S4.

An advanced mass spectrometric technique, CSI-FT-ICR-MS, was used to analyze the compositions of the precursor solutions, including La-only and Zr-only solutions as references, over the *m/z* range 0–4000. The CSI method ionizes molecules with minimum damage by cooling them down to the temperature of liquid nitrogen. The results are summarized in Fig. 3 and Table 2. The details of the compositional identification for the spectra are presented in Figure S4. The spectrum of the La-only solution shows clusters of the general formula La_n_(PrA)_3n_(PrAH)_x_, where PrA is the propionate ligand (CH_3_CH_2_COO) and n and x are integer numbers (n = 1–5 and x = 0–2 for the main clusters). The sodium ions found in some La clusters came from the glass parts used in the analyzer. These La clusters contained no acetic ligands, which were the ligands in the raw material (La acetate), indicating that La experienced a full ligand-exchange reaction with the solvent. The formula of the La clusters suggests that they were formed by the combination of single La(PrA)_3_ molecules and propionic acid molecules through van de Waals forces, without an oxide La–O–La core structure. They were most likely formed during spray ionization and solvent evaporation, during which some sodium ions were also incorporated into the clusters as contaminants. In the solution, because of the presence of a large amount of propionic acid solvent (La concentration was 0.2 mol/kg), La should exist in a single molecular form as La(PrA)_3_. In contrast, for the Zr solution, Zr6 clusters, having an oxide Zr–O–Zr core structure with 6 Zr atoms in each cluster, were identified in the m/z range 1440–1650, which was the dominating cluster size of Zr. The two strongest lines in the spectrum are attributed to Zr_6_O_5_(OH)_2_(PrA)_12_ and Zr_6_O_5_(OH)_3_(PrA)_11_, respectively. In addition, minor mass lines are found around m/z = 3000, which were estimated to be Zr clusters having 10–12 Zr atoms (Zr10~Zr12), whose exact composition could not be identified because of the high noise in the signals. This result is consistent with the reports that Zr and organic acids form Zr6 and Zr12 clusters, with the Zr12 cluster comprising two Zr6 clusters connected by organic ligands^23^,^29^.

The MS spectra of LZ37 solutions, both with and without solvothermal treatment, mainly showed a superposition of the La-only and Zr-only solutions, i.e., most La molecules and Zr clusters existed in the solution separately. Two discernable differences exist between LZ37 solutions and the La-only or Zr-only solution. First, the Zr12 clusters found in the Zr-only solution disappeared in the LZ37 solutions. Second, clusters that could be identified as La_2_Zr_5_O_6_(OH)_5_(PrA)_6_(Ac)_3_(H_2_O)_15_, containing both La and Zr atoms, were found in LZ37 solutions.

Based on the intensities of the MS lines, most of the detected La and Zr compounds separately existed in the mixed solutions of La and Zr, with only minor La–Zr cluster (i.e., clusters containing both La and Zr) content. However, this is inconsistent with the TG/DTA data, as well as with the HEXRD and XAFS analyses below, which can only be explained by the dominant presence of La–Zr clusters, and not by mechanically dispersed La-only and Zr-only clusters. We believe that the employed MS measurement system had low detection sensitivity for the La–Zr clusters. A possible reason for this is that the soft ionization method, i.e., cryospray ionization with liquid nitrogen cooling, has low ionization ability for the heavy La–Zr clusters. This consideration is supported by the much lower detection sensitivity observed for Zr6 clusters than for La clusters in the LZ37 solutions, as well as by the weak MS lines of the Zr10–Zr12 clusters, which are even two orders lower than those of the Zr6 clusters in the Zr-only solution (Fig. 3). Zr12 is believed to be the dominant cluster in our Zr-only solution, on the basis of previous single-crystal XRD studies which showed that Zr12 is the product in the same and similar solution systems^23^. Accordingly, we consider that La–Zr clusters were the dominant species present in the LZ37 solutions.

### HEXRD Analysis

**Figure 4:**
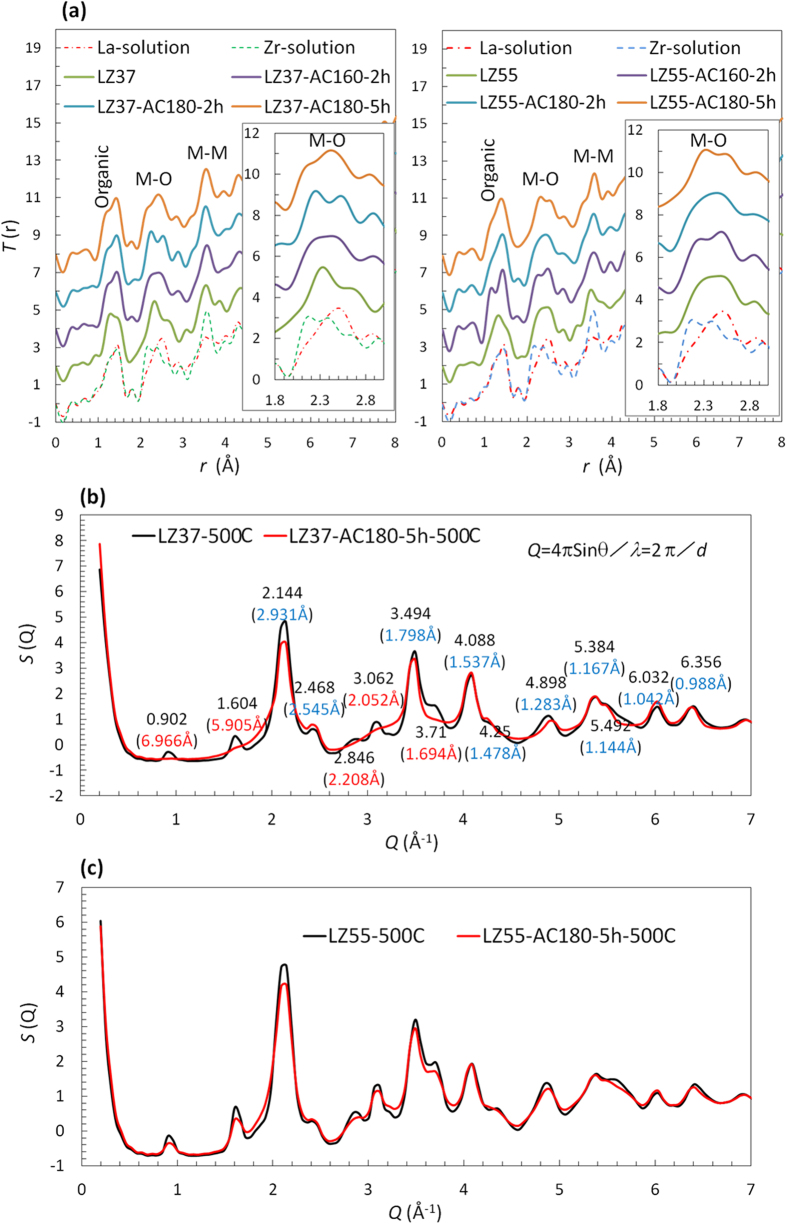
HEXRD analysis, showing the total correlation functions of solutions (**a**) and structure factors of powders annealed at 500 °C for LZ37 (**b**) and LZ55 (**c**). The insets in (**a**) show expanded plots of the metal–oxygen correlation. The *Q* values and the corresponding spacings (in parentheses) of the diffraction lines are indicated in (**b**).

Synchrotron high-energy XRD was performed to reveal the short- to medium-range structure of solutions, gels, and powders. The structure factor, *S*(*Q*), was derived from the raw data and then Fourier transformed to the pair distribution function, *G*(*r*), and total correlation function, *T*(*r*). Figure 4 displays typical data in which the differences between the samples with and without solvothermal treatment can be observed.

Figure 4(a) shows *T*(*r*) of solutions, where the data for the La-only and Zr-only solutions are also presented for reference. The difference in metal–oxygen (M–O) correlations between the samples is clearly observed. We first focus on the LZ37 solutions. Before solvothermal treatment, the co-existence of La and Zr in the solution led to an M–O correlation at a distance *r* = 2.3 Å in between the La–O and Zr–O peaks found in the reference La-only and Zr-only solutions. The side small peaks (*r* = 1.8 Å and 2.7–3.2 Å) may be artificial, arising from the truncation effect in the Fourier transformation. After solvothermal treatment at 180 °C for 2 h, the M–O peak split into two. After a longer solvothermal treatment at 180 °C for 5 h, both peaks decreased and one peak grew between them. In LZ55 solutions, the tendency (one peak → splitting into two → decreasing of both peaks and growth of another in between) was the same but the relative peak intensities were different. These changes in M–O correlations can be explained as follows. Before solvothermal treatment, La–Zr clusters containing both La and Zr had formed, which were subject to structural reorganization during solvothermal treatment to form a stable structure. This is consistent with the above TG/DTA analysis.

The effect of solvothermal treatment of the solutions on the final material structure is clearly observed in the HEXRD data. Figure 4(b,c) show *S*(*Q*) for powders annealed at 500 °C, and Table 3 lists the plane spacing values calculated from *S*(*Q*) of the LZ37 samples alongside the values of cubic ZrO_2_ (ref. 27). Note that the samples had a cubic structure, though the regular laboratory XRD and TEM suggested an amorphous state, indicating that the atoms were only locally ordered. In addition to the cubic structure, an unidentified minor phase was found in the LZ37 powder obtained from the precursor solution without solvothermal treatment, which almost vanished in the powder obtained from the solvothermally treated solution. For the LZ55 samples, solvothermal treatment of the solution only resulted in a decreased amount of the minor phase, not its elimination. Therefore, the minor phase was considered to be a La-rich phase.

This result undoubtedly proves that solvothermal treatment led to a uniform structure in the precursor, which was even inherited by the final annealed oxide. Without pre-formation of a uniform cluster structure, the final material remained non-uniform even if the metal components in the precursors were well mixed on the cluster scale, but not inside the clusters. This suggests that the inorganic core of the cluster in the precursor remained the structural unit after annealing, during which the organic ligands around it decomposed and the cores compacted, without significantly reacting with the surrounding components, such as the La-rich phase.

### XAFS Analysis

**Figure 5:**
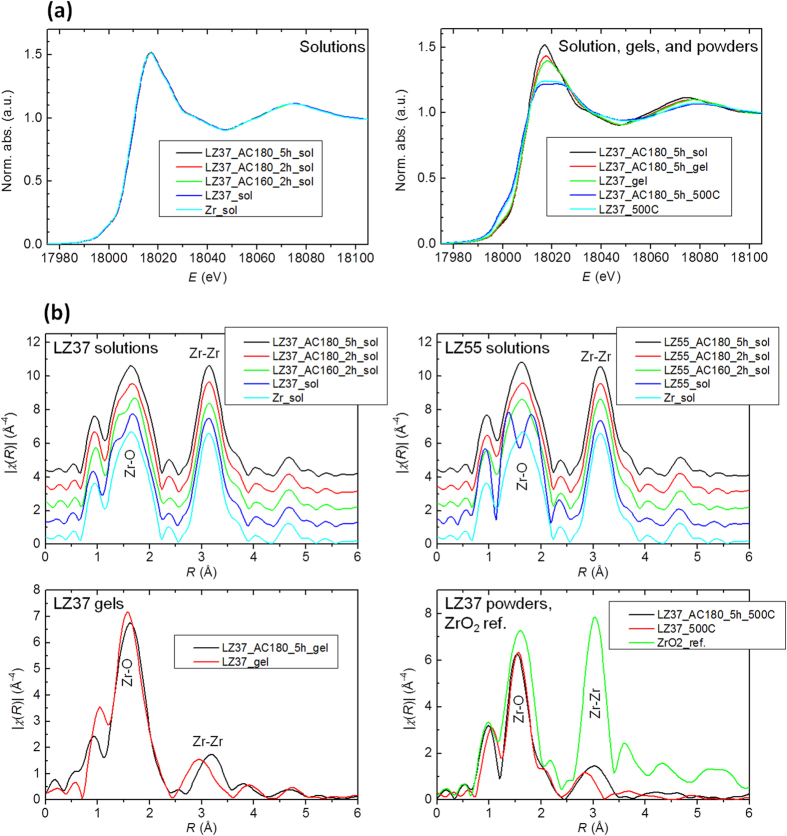
XAFS analysis, showing the XANES data (**a**) and the FT-XAFS data (**b**) at the Zr *K* edge.

XAFS analysis was used to quantitatively probe the local atomic structure, specifically the structure of the nearest and second-nearest atoms around a selected atom. Data for absorptions at the La-K and Zr-K edges were recorded for solutions, gels, and powders. The XANES and EXAFS data at the La-K edge (Figure S5) showed no convincing differences for all samples, though that solvothermal treatment of the LZ37 solution resulted in a very slightly increased amplitude of the La–O peak in the Fourier-transformed XAFS spectra (also regarded as the radial structural function), which possibly indicate an very small increase in the number of O atoms next to La. These data showed clearly only the nearest atoms (O next to La), but not the second-nearest atoms, i.e., La(Zr) in La–O–La(Zr). This may be attributed to a weak signal at the La-K edge, resulting in qualified data only up to *k* = 9 Å^−1^ (Figure S5). In contrast, the results at the Zr-K edge (Fig. 5) clearly show the effects of solvothermal treatment, which we present in detail below.

The XANES spectra at the Zr-K edge (Fig. 5(a)) give qualitative information about the Zr–O polyhedra or the nearest O to the Zr^30^,^31^. The shape of the white line reflects the number of O atoms in the polyhedra. The white lines for all the LZ solutions are the same, having a broad single band corresponding to a coordination number (CN) of 7 or more for O atoms around Zr atoms. This is consistent with the Zr6 cluster-based structures found in the MS analysis. Such Zr6 clusters have eight-fold coordination of O around Zr^23^. For powders annealed at 500 °C, the white lines split into two bands, indicating an octahedral (CN ~ 6) Zr–O polyhedron, which seems, however, inconsistent with the cubic structure found in the above HEXRD analysis, as the CN of Zr–O polyhedra in an ideal cubic ZrO_2_ structure is 8. Therefore, the powders had a cubic structure that deviates from the ideal one, having ~6 O atoms next to each Zr atom. The solvothermal treatment resulted in more splitting of the white line. For gel samples, the shape of the white line lies between those of the solutions and the annealed powders, suggesting an intermediate coordination state of O around Zr. In addition, a clear shoulder at 18000 eV (before the edge jump) in the XANES spectra of the powders indicated an evident distortion from centrosymmetric Zr–O polyhedra^32^. This again indicates that the powder deviates from the ideal 8-fold Zr–O coordination. The solutions also showed a shoulder at 1800 eV at much reduced intensity, indicating a slight distortion from centrosymmetric Zr–O polyhedra. The effect of solvothermal treatment was not clear in this shoulder of XANES spectra.

The FT-XAFS spectra presented in Fig. 5(b) clearly indicates that the shape of the Zr–O band for solutions exhibits an apparent dependence on solvothermal treatment, and the Zr–Zr distances in gels and powders are considerably increased by the treatment.

**Figure 6:**
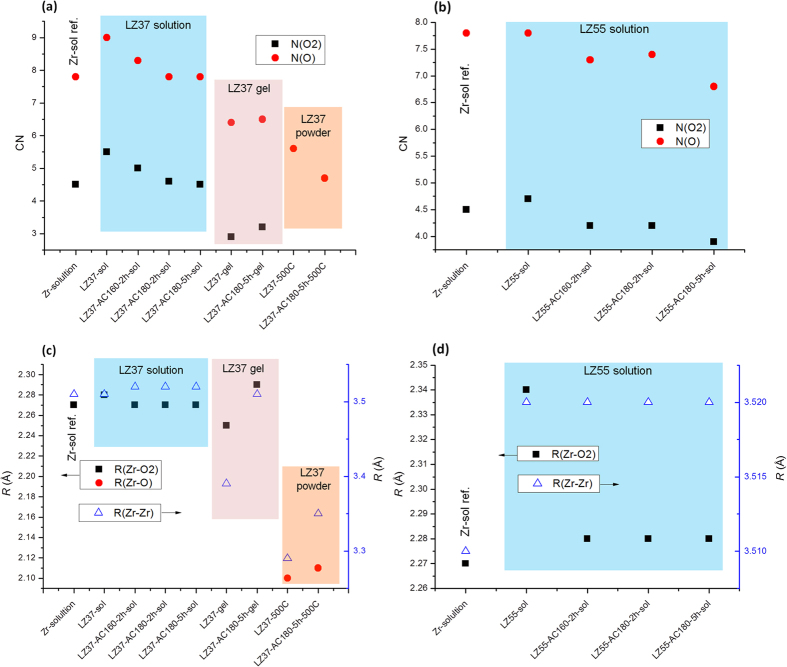
Fitting results of the XAFS data, showing the coordination numbers and interatomic distances of the LZ37 ((**a**,**c**), respectively) and LZ55 ((**b,d**), respectively) samples.

Data fitting was performed to obtain quantitative CN and interatomic distance values. On the basis of the above analyses, a structural model based on Zr6 clusters (Figure S2(a)) was used for solutions and gels, and a cubic ZrO_2_ model (Figure S2(b)) was used for powders. In the structure of Zr6 clusters, the Zr–O distance varies with the variation of the local environment (Zr-O-Zr, Zr-O-H, Zr-O-COCH_2_CH_3_, and geometry; see refs 23, 29 for details). In the data fitting, we divided the oxygen in Zr-O bonds into two groups (O1 and O2, at relatively short and long distances, respectively, from Zr). The results are listed in Table 4 and the effects of solvothermal treatment seen in these results are summarized in Fig. 6, where N(O) represents the total CN of O1 and O2 (N(O) = N(O1) + N(O2)) for solutions and gels, or the CN of O around a Zr atom for powders.

The CNs of LZ37 solutions, gels, and annealed powders are displayed in Fig. 6(a). The variation in the total CNs of O was mainly contributed by the change in the CNs of O2. In the LZ37 solutions, the CN of O was larger than that of Zr-only solutions (CN ~8, consistent with a Zr6 cluster) before solvothermal treatment. After solvothermal treatment, the CN of O gradually decreased and finally became similar to that of the Zr-only solution. Considering the large uncertainties of around ±1.0 in the calculated CN values, a comparison between only two CN values with a difference of less than 1.0 may be invalid; however, the overall tendency shown by several values is considered not to be caused by uncertainties. The variation in CN values again suggests that the La and Zr compounds were not just mechanically mixed. Rather, they formed an intermediate structure upon mixing, which was re-structured into stable La–Zr clusters during solvothermal treatment. In the gels and powders, solvothermal treatment did not change the CN of O within the calculation uncertainty, though it possibly reduced the CN in the powders (by 0.9).

In the LZ55 solutions, the CN of O even decreased to below 7 after solvothermal treatment (Fig. 6(b)). This can be explained by more incorporation of La atoms in the La–Zr cluster, which leads to less oxygen because the valence state of La (3+) is lower than that of Zr (4+).

By comparing the CNs in solutions, gels, and powders, it is apparent that the CNs of O in solutions are the largest, those in powders are the smallest, and those in gels are in the middle. This suggests that the gels had a structure between those of the solutions and powders. The CNs of O in solutions were consistent with that in a Zr–carboxylate cluster (Zr6-based, CN of O = 8)^23^, whereas those in powders (5.6 and 4.7 for solutions before and after solvothermal treatment, respectively) were substantially smaller than the ideal value (8) in a cubic ZrO_2_ structure. This is consistent with the above XANES observations. The small CN of O in the powders is attributed to the inclusion of a large amount of La.

The calculated interatomic distances in LZ37 solution, gel, and powder are plotted in Fig. 6(c), while those in the LZ55 solution are displayed in Fig. 6(d). In contrast to the CN values, the distance values were relatively precise, typically within ±0.01 to ±0.02 Å.

First, we examined the interatomic distances in solutions. Solvothermal treatment led to decreased Zr–O2 distances in solutions, which is not considered to arise from calculation uncertainties since the tendency is consistently observed in both LZ37 and LZ55 solutions. The addition of La also increased the distance. In the LZ37 solution, where La/Zr = 3/7, the presence of La only led to a slight increase (0.01 Å) in the Zr–O2 distance before solvothermal treatment. In contrast, in the LZ55 solution, where La/Zr = 5/5, this increase was much larger (0.06 Å). Further, in the LZ37 solution, this distance was reduced after solvothermal treatment to the same value as that in the Zr-only solution, while in the LZ55 solution, it was still longer than that in the Zr-only solution after the treatment. These variations in Zr–O2 point to the formation of an unstable La–Zr cluster structure with a longer Zr–O2 distance than that in the Zr6 before the solvothermal treatment, which re-organized into a stable structure with a reduced Zr–O2 distance during solvothermal treatment. It is reasonable that the increased La content led to more structural deviation from the Zr cluster. Solvothermal treatment or the presence of La appears to slightly increase the Zr–Zr distance from 3.51 Å to 3.52 Å. The consistent tendencies of the data in both LZ37 and LZ55 solutions suggest that this is not likely to be a result of the data uncertainty. In the LZ55 solution, the Zr-Zr distance reached 3.52 Å even before solvothermal treatment. This suggests that solvothermal treatment, as well as the addition of more La, led to more La incorporation into the multi-nuclear clusters (the ionic radius of La is larger than that of Zr: 117 pm for La^3+^ and 86 pm for Zr^4+^).

Next, we examined the interatomic distances in gels and powders. We found that solvothermal treatment led to increases in both Zr–O2 (increased from 2.25 Å to 2.29 Å) and Zr–Zr (from 3.39 Å to 3.51 Å) distances in gels, and in Zr–Zr distance (from 3.29 Å to 3.35 Å) in powders. This again suggests that solvothermal treatment led to more La incorporation into the clusters. The observed increase in Zr–O distance in powders was within the calculation uncertainty and thus could not be confirmed without the measure of more samples to clarify the tendency.

Comparing the interatomic distances of solutions, gels, and powders, we note that the values for solutions were the largest, those for the powders were the smallest, and those for the gels were in the middle. This again indicates that the gels had a structure between those of the solutions and powders, as suggested by the above CN analysis. The interatomic distances in solutions were consistent with the values for Zr6-based clusters (around 2.1–2.3 Å for Zr–O and around 3.5 Å for Zr–Zr)^30^. As for the distance values for powders, the Zr–O values were similar to that in a cubic ZrO_2_ crystal (2.15 Å), whereas the Zr–Zr values (3.29 Å and 3.35 Å) were much smaller than that in a cubic ZrO_2_ crystal (3.55 Å). This can be attributed to the lack of high ordering in our powders, which is also supported by much smaller CNs of O next to Zr (5.6 and 4.7) and of Zr next to a second Zr (i.e., second-nearest neighbor, less than 5) than those in a cubic ZrO_2_ crystal, where the CNs of Zr–O and Zr–Zr are 8 and 12, respectively. Thus, the local ordering was restricted to within approximately the second-nearest neighboring atoms, corresponding to a diameter of ~7 Å.

### Analysis of Elemental Composition for the Annealed Films

In order to investigate the effect of solvothermal treatment on the elemental composition of the annealed films, precise compositional analysis approaches (RBS/HFS/NRA, with estimated precisions of ±0.5 atom% for C and H, ±1 atom% for La and Zr, and ±4 atom% for O) developed for thin-film samples were applied as described in the Experimental Methods section.

The results are tabulated in Table 5. The solvothermal treatment led to increased O content in the films. Taking normal chemical states of La, Zr, and O ions as 3+, 4+, and 2−, respectively, the charges of O ions were balanced by those of La and Zr ions in the samples obtained from solutions without solvothermal treatment. In contrast, in the samples obtained from solutions with solvothermal treatment, the charges of O ions could not be balanced only by those of La and Zr ions. Instead, charge balance could only be achieved by including C and H in the 4+ and 1+ oxidation states, respectively. This suggests that the residual carbon in the films obtained from solutions without solvothermal treatment was not bonded to oxygen, whereas that in films obtained from solutions with solvothermal treatment was bonded to oxygen. This is similar to the results obtained for the low-temperature annealed films we will report in another paper^20^, which are consistent with the XPS data. Hence, in the former case (without solvothermal treatment), the solid was likely to consist of M–O polyhedra and either free C or C bonded to H (organic C). In addition to the La-rich second phase, such C further increased the heterogeneity of the solid structure. In the latter case, both C and H were bonded to O and likely to be incorporated in the network structure of the amorphous solid, although H might also exist on the surface in the form of M–O–H groups. Accordingly, the structure of the solid was expected to be mainly composed of a network containing M–O polyhedra and C–O polyhedral, which are more likely to form a homogeneous network structure than the former case. Because the C and H structures were so different in the two cases, the insulating properties of the films were significantly lower in the former case than in the latter. Probing the location of C in the structure will be the subject of later studies.

### Summary of the Above Analyses

The analyses described above are summarized as follows:

- The thermal behavior of the La–Zr precursor different from those of the La-only and the Zr-only ones in TG/DTA analysis suggested formation of La–Zr clusters (containing both La and Zr in one cluster) in the precursor. The TG/DTA data also indicated that the clusters developed toward a more unified and more stable structure with solvothermal treatment. HEXRD and XAFS analyses provided further support for the formation of La–Zr clusters by showing a structure of the La–Zr precursor different from both the La-only and Zr-only ones. The HEXRD and XAFS data also allowed detailed analysis for the short range structure. The MS analysis appeared to have a low relative sensitivity for the Zr-containing clusters but also indicated formation of La–Zr clusters.
- La-only and Zr-only solutions: La did not form clusters with La–O–La cores. It essentially existed as La(PrA)_3_ molecules in the solution. Zr formed Zr6 and approximately Zr10–Zr12 clusters.
- La–Zr solutions before solvothermal treatment: Zr10–Zr12 clusters vanished, La–Zr clusters formed, and Zr6 and La molecules continued to exist, but should be minor compounds according to all data apart from MS.
- La–Zr solutions after solvothermal treatment: The La–Zr clusters re-structured toward structural unification, forming more stable and more uniform clusters with increased La incorporation and reduced organic ligand content. The CNs and interatomic distances were correspondingly altered. The clusters tended to form a single-type structure.
- Annealed solids: The final oxide was only locally ordered, possessing a cubic structure that had significantly small CNs of O atoms in Zr–O polyhedra with non-centrosymmetric geometry, as well as significantly small CNs of the second-nearest Zr atoms. The local ordering had a diameter of ~7 Å. Solvothermal treatment of the solution led to an increased Zr–Zr distance, which is attributed to more La inclusion, accompanied by vanishing of a second La-rich phase. For LZ55, the content of La was too high to be wholly included in the La-Zr clusters, and thus, the second La-rich phase remained. The carbon atoms in the films from the solvothermally treated solutions appeared to be bonded with O, while those in the films from the solution without solvothermal treatment appeared to be in a free or H-bonded state.
- Gels: The gels had structures between those of solutions and powders.

## Conclusions

In the solutions of lanthanum acetate and zirconium butoxide in propionic acid (PrA), La existed as La(PrA)_3_ molecules without a La–O–La core in the La-only solution, while Zr was present as multi-nuclear clusters (Zr6 and estimated Zr10–Zr12) with Zr–O–Zr cores in the Zr-only solution. When the two solutions were mixed, the La and Zr structures did not simply mechanically mix. Instead, they formed clusters in which La and Zr coexisted. Solvothermal treatment of the solution led to changes toward structural unification in the La–Zr clusters, leading to higher stability and higher uniformity with more La incorporation, accompanied by corresponding changes in CNs and interatomic lengths of nearest (Zr–O) and second-nearest (Zr–Zr) neighbors. Therefore, the solvothermally treated La–Zr solutions resulted in uniform oxide solids with a reduced, or even removed, La-rich second phase. Because the structure of the cores of the La–Zr clusters in solution determined the structure of the final oxide solids, the solvothermally treated solutions led to films with improved insulating and dielectric properties. The re-organization of the clusters under solvothermal conditions also enhanced UV light absorption and enabled film deposition under UV light at low temperatures.

The LaZr oxide solids were amorphous with local ordering of a cubic ZrO_2_ structure, but had significantly fewer O atoms (~6 or less) next to the Zr atoms, as compared to usual cubic ZrO_2_ crystals (8 O atoms). The second-nearest Zr atoms were even fewer (below 5) than those in a cubic ZrO_2_ crystal (12), indicating that the local ordering was restricted to within the second-nearest neighboring atoms, with a diameter of ~7 Å. The carbon atoms in films obtained from solvothermally treated precursor solutions appeared to be bonded with O, while those in the films obtained from untreated solutions appeared to be in a free or H-bonded state, which could also be a reason for the differences in their insulating properties.

## Additional Information

**How to cite this article**: Li, J. *et al*. Hybrid Cluster Precursors of the LaZrO Insulator for Transistors: Properties of High-Temperature-Processed Films and Structures of Solutions, Gels, and Solids. *Sci. Rep*. **6**, 29682; doi: 10.1038/srep29682 (2016).

## Supplementary Material

Supplementary Information

## Figures and Tables

**Table 1:** Densities (in g cm^−3^) of the LaZrO films.

	LZ37	LZ37-AC180-2 h	LZ37-AC180-5 h	LZ55	LZ55-AC180-5 h
400 °C	4.31 (70.7%)	4.46 (73.1%)	4.73 (77.5%)	4.75 (77.9%)	5.15 (84.4%)
500 °C	4.74 (77.7%)	4.94 (81.0%)	4.97 (81.5%)	4.87 (79.8%)	—
600 °C	4.77 (78.2%)	4.93 (80.8%)	5.15 (84.4%)	5.24 (85.9%)	5.47 (89.7%)

Values in parentheses are the densities relative to a single crystal.

**Table 2:** Assignment of the MS data for the La structures.

Meas. monoisotopic mass (examples)	Structure	Calc. monoisotopic mass
433.0375	La(PrA)_3_(PrAH)H^+^	433.0378
507.0741	La(PrA)_3_(PrAH)_2_H^+^	507.0746
716.9940	La_2_(PrA)_6_H^+^	716.9943
791.0305	La_2_(PrA)_6_(PrAH)H^+^	791.0310
865.0678	La_2_(PrA)_6_(PrAH)_2_H^+^	865.0678
1074.9879	La_3_(PrA)_9_H^+^	1074.9875
1149.0243	La_3_(PrA)_9_(PrAH)H^+^	1149.0242
1432.9813	La_4_(PrA)_12_H^+^	1432.9807
1507.0184	La_4_(PrA)_12_(PrAH)H^+^	1507.0175
1791.0077	La_5_(PrA)_15_H^+^	1790.9739
Examples of H → Na		
551.0384	La(PrA)_3_(PrAH)(PrANa)Na^+^	551.0385
813.0127	La_2_(PrA)_6_(PrAH)Na^+^	813.0130
834.9948	La_2_(PrA)_6_(PrANa)Na^+^	834.9949

PrA = C_2_H_5_COO; PrAH = C_2_H_5_COOH.

**Table 3:** Comparison of the HEXRD data with the XRD data of cubic zirconium oxide.

LZ37-500C	LZ37-AC180-5 h-500C	Cubic Zr_0.9_La_0.1_O_1.95_ (ref. 27)		
*d* (Å)	*d* (Å)	*d* (Å)	hkl	Intensity
6.966	X			
5.905	X			
2.931	2.931	2.9531	111	100
2.545	2.545	2.5575	200	21
2.208	X			
2.052	X			
1.798	1.798	1.8084	220	48
1.694	X			
1.537	1.537	1.5422	311	30
1.478	1.478	1.4765	222	4.5
1.283	1.283	1.2787	400	5.2
1.167	1.167	1.1734	331	9.4
1.144	1.144	1.1437	420	4.9

**Table 4:** Fitting results for the XAFS data.

Sample	Abs-Bs	N(Bs)	N(O1+O2)	R (Å)	σ^2^ (Å^2^)	R-factor (%)
Zr-sol	Zr-O1	3.3 ± 0.5	7.8	2.11 ± 0.02	0.004 ± 0.002	0.03
	Zr-O2	4.5 ± 0.8		2.27 ± 0.02		
	Zr-Zr1	3.3 ± 1.1		3.51 ± 0.01	0.005 ± 0.002	
LZ37-sol	Zr-O1	3.5 ± 0.5	9.0	2.12 ± 0.02	0.007 ± 0.002	0.01
	Zr-O2	5.5 ± 1.1		2.28 ± 0.02		
	Zr-Zr1	3.1 ± 0.7		3.51 ± 0.01	0.0046 ± 0.0009	
LZ37-AC160-2 h-sol	Zr-O1	3.3 ± 0.5	8.3	2.12 ± 0.02	0.0055 ± 0.0024	0.02
	Zr-O2	5.0 ± 1.0		2.27 ± 0.02		
	Zr-Zr1	3.4 ± 1.0		3.52 ± 0.01	0.0051 ± 0.0013	
LZ37-AC180-2 h-sol	Zr-O1	3.2 ± 0.5	7.8	2.11 ± 0.02	0.004 ± 0.002	0.02
	Zr-O2	4.6 ± 0.8		2.27 ± 0.02		
	Zr-Zr1	3.4 ± 1.0		3.52 ± 0.01	0.005 ± 0.001	
LZ37-AC180-5 h-sol	Zr-O1	3.3 ± 0.5	7.8	2.12 ± 0.02	0.004 ± 0.002	0.03
	Zr-O2	4.5 ± 0.8		2.27 ± 0.02		
	Zr-Zr1	3.4 ± 1.1		3.52 ± 0.01	0.005 ± 0.002	
LZ37-gel	Zr-O1	3.5 ± 1.8	6.4	2.12 ± 0.04	0.006 ± 0.006	0.12
	Zr-O2	2.9 ± 1.1		2.25 ± 0.02		
	Zr-Zr1	10 ± 10		3.39 ± 0.03	0.02 ± 0.01	
LZ37-AC180-5 h-gel	Zr-O1	3.3 ± 0.5	6.5	2.14 ± 0.02	0.0039 ± 0.0018	0.02
	Zr-O2	3.2 ± 0.4		2.29 ± 0.02		
	Zr-Zr1	3.3 ± 1.7		3.51 ± 0.03	0.013 ± 0.004	
LZ37-500C	Zr-O1	5.6 ± 1.4		2.10 ± 0.02	0.010 ± 0.003	0.4
	Zr-Zr1	4.9 ± 3.8		3.29 ± 0.02	0.019 ± 0.004	
LZ37-AC180-5 h-500C	Zr-O1	4.7 ± 0.9		2.11 ± 0.02	0.0095 ± 0.0022	0.3
	Zr-Zr1	2.2 ± 2.6		3.35 ± 0.07	0.010 ± 0.007	
LZ55-sol	Zr-O1	3.1 ± 0.5	7.8	2.07 ± 0.01	0.002 ± 0.001	0.03
	Zr-O2	4.7 ± 0.8		2.34 ± 0.01		
	Zr-Zr1	3.8 ± 1.3		3.52 ± 0.01	0.006 ± 0.002	
LZ55-AC160-2 h-sol	Zr-O1	3.1 ± 0.7	7.3	2.12 ± 0.02	0.004 ± 0.002	0.02
	Zr-O2	4.2 ± 1.0		2.28 ± 0.02		
	Zr-Zr1	3.4 ± 1.1		3.52 ± 0.01	0.005 ± 0.002	
LZ55-AC180-2 h-sol	Zr-O1	3.1 ± 0.5	7.4	2.12 ± 0.02	0.004 ± 0.002	0.02
	Zr-O2	4.3 ± 0.9		2.28 ± 0.02		
	Zr-Zr1	3.4 ± 1.0		3.52 ± 0.01	0.005 ± 0.001	
LZ55-AC180-5 h-sol	Zr-O1	2.9 ± 0.7	6.8	2.13 ± 0.03	0.003 ± 0.002	0.04
	Zr-O2	3.9 ± 1.0		2.28 ± 0.03		
	Zr-Zr1	3.4 ± 1.4		3.52 ± 0.02	0.005 ± 0.002	

Abs = X-ray absorbing atom. Bs = backscatterer. N = coordination number. R = interatomic distance Abs-Bs. σ^2^ = Debye-Waller factor. R-factor = quality of fit.

**Table 5:** Elemental composition of the LaZrO films.

	Measured	Calculated O, I^b^	Calculated O, II^c^
LZ37, 400 °C	La_0.39_ZrO_2.71_C_0.29_H_0.51_	La_0.39_ZrO_2.60_	La_0.39_ZrO_3.42_C_0.29_H_0.51_
LZ37-AC180-5h, 400 °C	La_0.40_ZrO_3.26_C_0.24_H_0.60_	La_0.40_ZrO_2.60_	La_0.40_ZrO_3.38_C_0.24_H_0.60_
LZ55, 600 °C	La_0.82_ZrO_3.22_C_0.11_H_0.53_	La_0.82_ZrO_3.23_	La_0.82_ZrO_3.72_C_0.11_H_0.53_
LZ55-AC180-5 h, 600 °C	La_0.87_ZrO_4.05_C_0.16_H_0.23_	La_0.87_ZrO_3.31_	La_0.87_ZrO_3.74_C_0.16_H_0.23_

^a^The calculation of O was based on the charge-neutral condition, assuming the states of La 3+, Zr 4+, C 4+, and H 1+.

^b^Calculated O contents without the consideration of C and H.

^c^Calculated O contents with the consideration of C and H.
