# Supplementary material for: Hybrid Cluster Precursors of the LaZrO Insulator for Transistors: Properties of High-Temperature-Processed Films and Structures of Solutions, Gels, and Solids
Source: Sci Rep. 2016 Jul 14;6:29682. doi: 10.1038/srep29682 (PMC4944196; doi:10.1038/srep29682)
Supplement: Supplementary Information [file srep29682-s1.pdf]

Hybrid Cluster Precursors of the LaZrO Insulator for Transistors: Properties of High-Temperature-Processed Films and Structures of Solutions, Gels, and Solids

Jinwang Li,[1,2]* Peixin Zhu,[1,3] Daisuke Hirose,[1,4] Shinji Kohara,[4,5,6] Tatsuya Shimoda[1,2,4]

[1] Green Device Research Center, Japan Advanced Institute of Science and Technology (JAIST), 2-13 Asahidai, Nomi, Ishikawa 923-1211, Japan

[2] Japan Science and Technology Agency (JST), ERATO, Shimoda Nano-Liquid Process Project, 2-13 Asahidai, Nomi, Ishikawa 923-1211, Japan

[3] Core Functionalities Development Center, Central Research Laboratories, DIC Corporation, 631 Sakado, Sakura, Chiba 285-8668, Japan

[4] School of Materials Science, Japan Advanced Institute of Science and Technology (JAIST), 1-1 Asahidai, Nomi, Ishikawa 923-1292, Japan

[5] SPring-8/Japan Synchrotron Radiation Research Institute (JASRI), 1-1-1 Kouto, Sayo-cho, Sayo-gun, Hyogo 679-5198, Japan

[6] Synchrotron X-Ray Group, Quantum Beam Unit, National Institute for Materials Science, 1-1-1 Kouto, Sayo-cho, Sayo-gun, Hyogo 679-5148, Japan

* Corresponding author. Email: lijw@jaist.ac.jp.

Supplementary Information

Details of the calculation of γ

We define γ as the mass ratio of oxygen to metals in a pure LaZrO oxide, assuming oxidation states of La 3+, Zr 4+, and O 2-. For an oxide with a La/Zr ratio of 3/7, under charge-neutral conditions, the composition is $La_{0.3}Zr_{0.7}O_{1.85}$, which leads to

$$\gamma = 16.00 \text{ g/mol} \times 1.85 \text{ mol} / (138.9 \text{ g/mol} \times 0.3 \text{ mol} + 91.22 \text{ g/mol} \times 0.7 \text{ mol}) = 0.28$$

where 16.00, 138.9, and 91.22 (in g/mol) are the masses of O, La, and Zr, respectively.

(a)

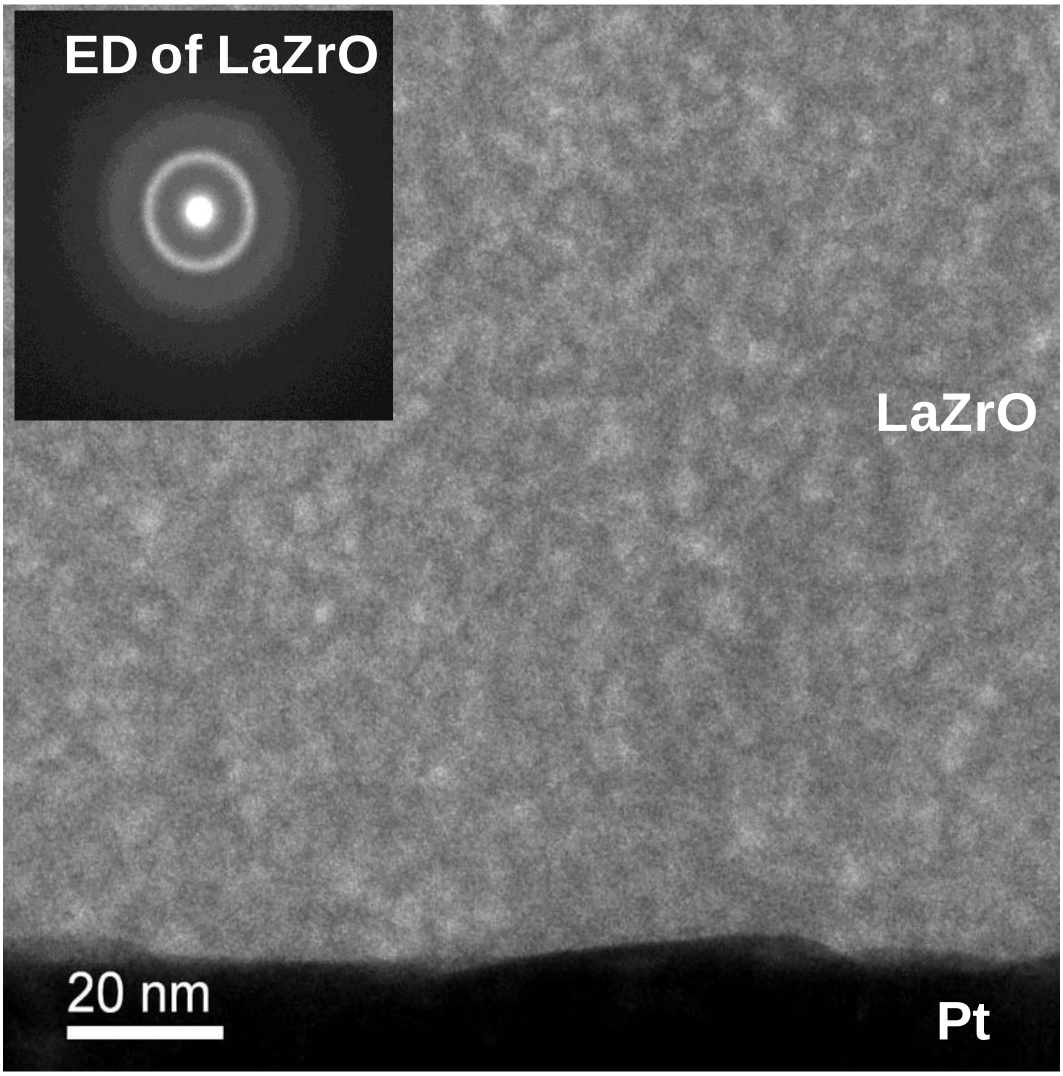

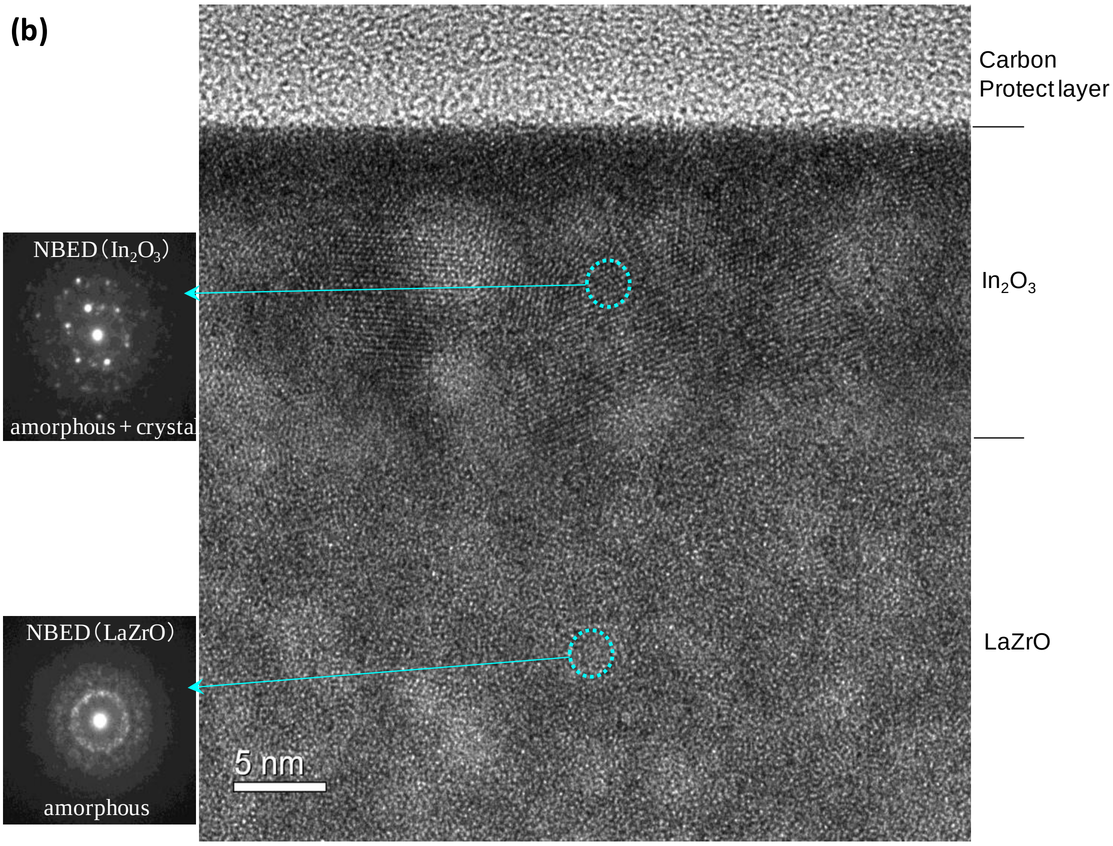

Figure S1. (a) TEM image and electron diffraction (ED) pattern of a LaZrO film (La/Zr molar ratio = 3/7) annealed at 400 °C. The preparation of the sample was the same as described in refs. 18 and 19 and in the Experimental Methods section of this paper (without solvothermal treatment of the precursor solution). (b) TEM image of the LaZrO at a higher resolution and its nano-beam electron diffraction (NBED) patterns. An indium oxide (In_2O_3) layer with nanocrystalline structure was deposited on the LaZrO for comparison. The diffracted areas are marked by circles. The image and NBED patterns indicate that the In_2O_3 was composed of crystalline and amorphous phases while the LaZrO was composed of an amorphous phase.

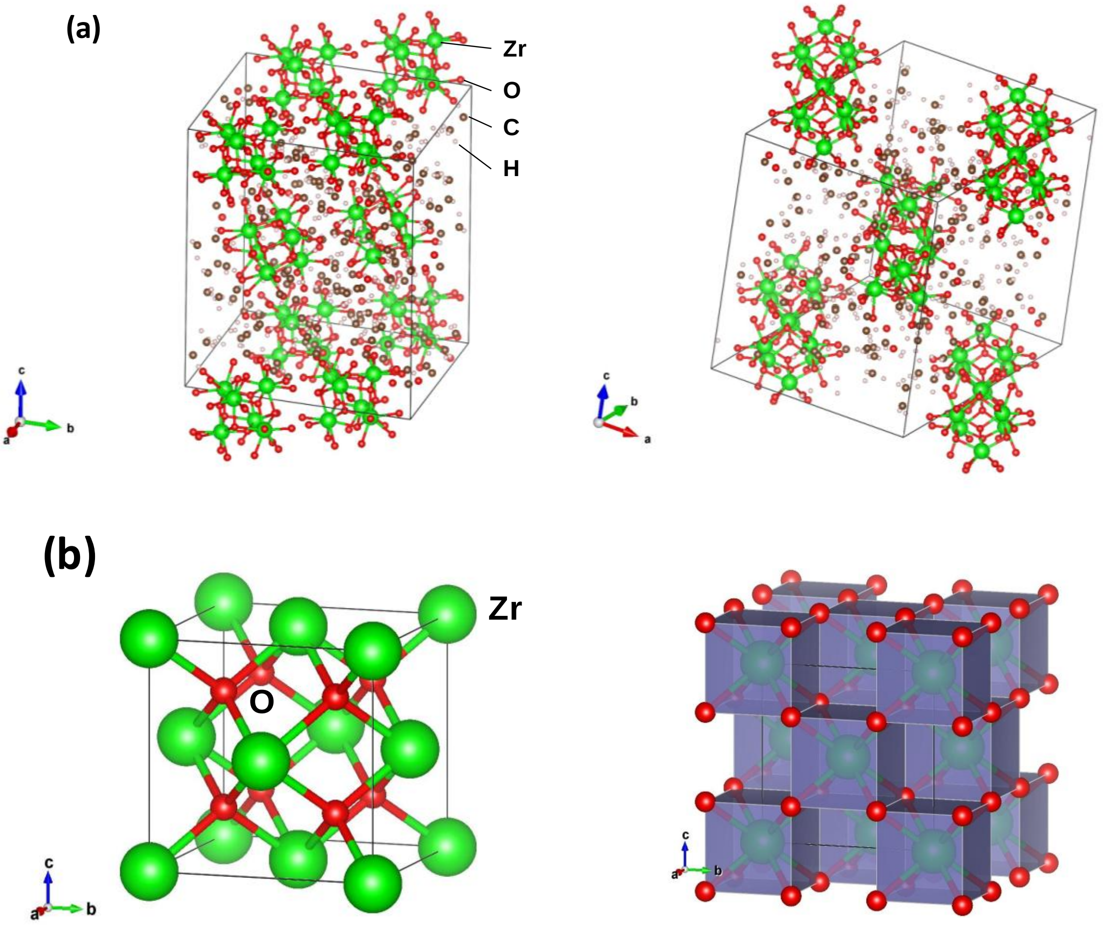

Figure S2. (a) The structure of the Zr12 cluster, $[Zr_6O_4(OH)_4(OOCR)_{12}]_2$, viewed from two different directions. The oxygen atoms bonded to a Zr atom are better visible in the left panel, whereas each Zr12 cluster is better distinguishable in the right panel. The Zr, O, C, and H atoms are shown, but only the Zr–O bonds are indicated for clarity. The Zr12 cluster comprises two Zr6 clusters, $Zr_6O_4(OH)_4(OOCR)_{12,}$ bridged by carboxylate ligands. In this figure, the ligands are propionate. (b) Crystal structure of cubic ZrO_2. The ZrO_8 polyhedra are shown in the right panel. This figure was drawn using the software VESTA (ref. S1) according to the published crystallographic data (ref. S2 for (a) and ref. S3 for (b)).

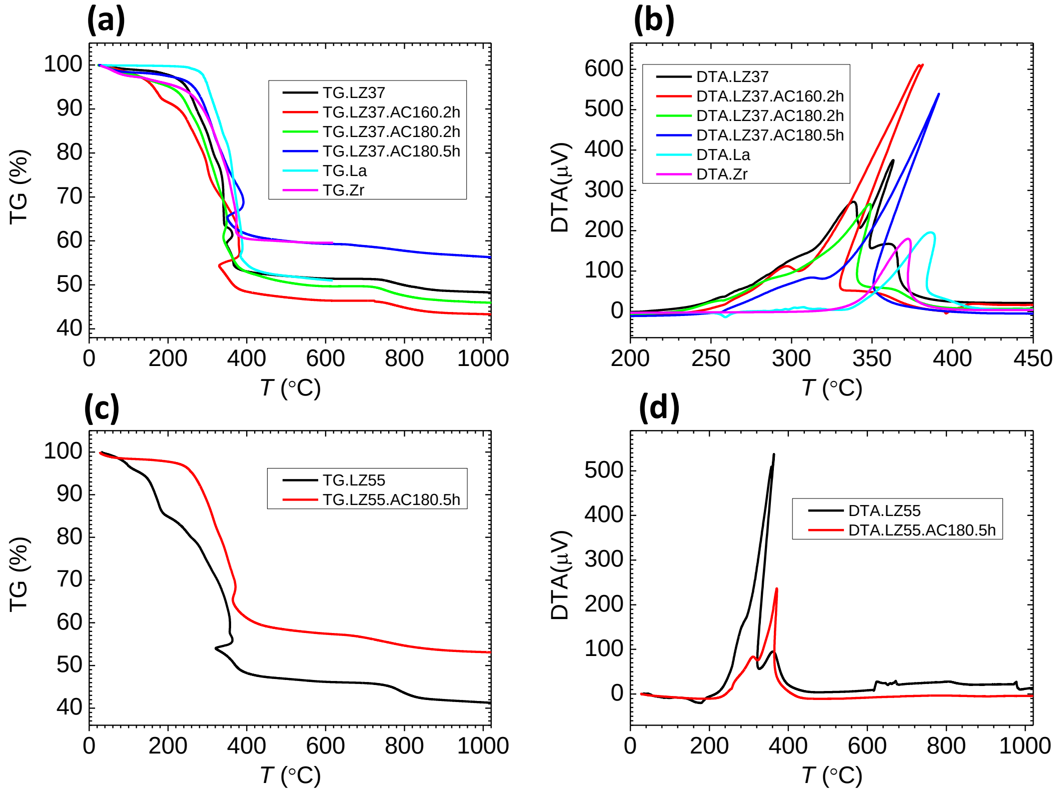

Figure S3. TG ((a) and (c)) and DTA ((b) and (d)) data for the precursor gels of the LZ37 ((a) and (b)) and LZ55 ((c) and (d)) samples, plotted against the sample temperature.

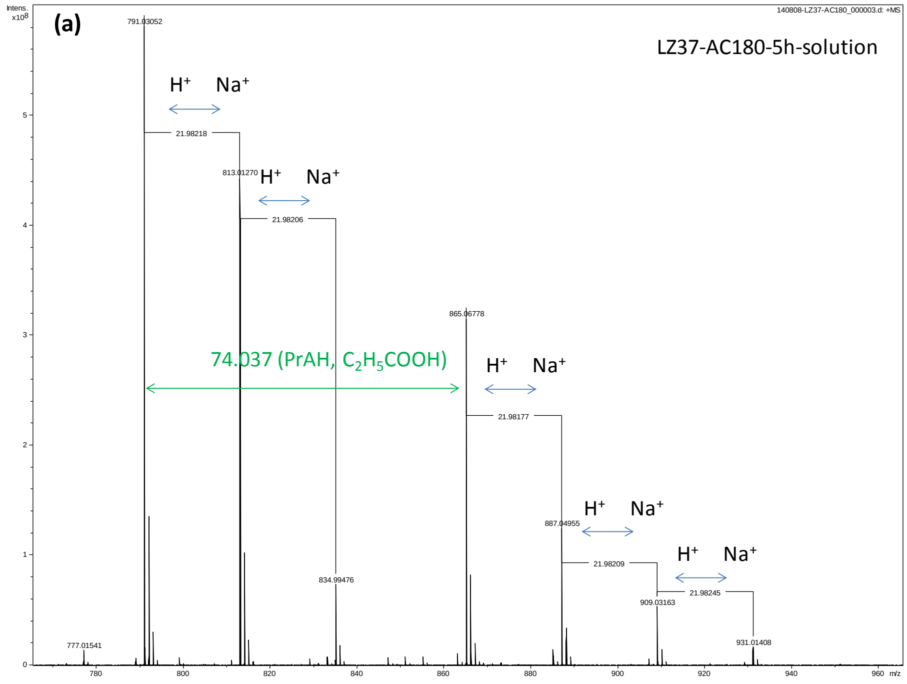

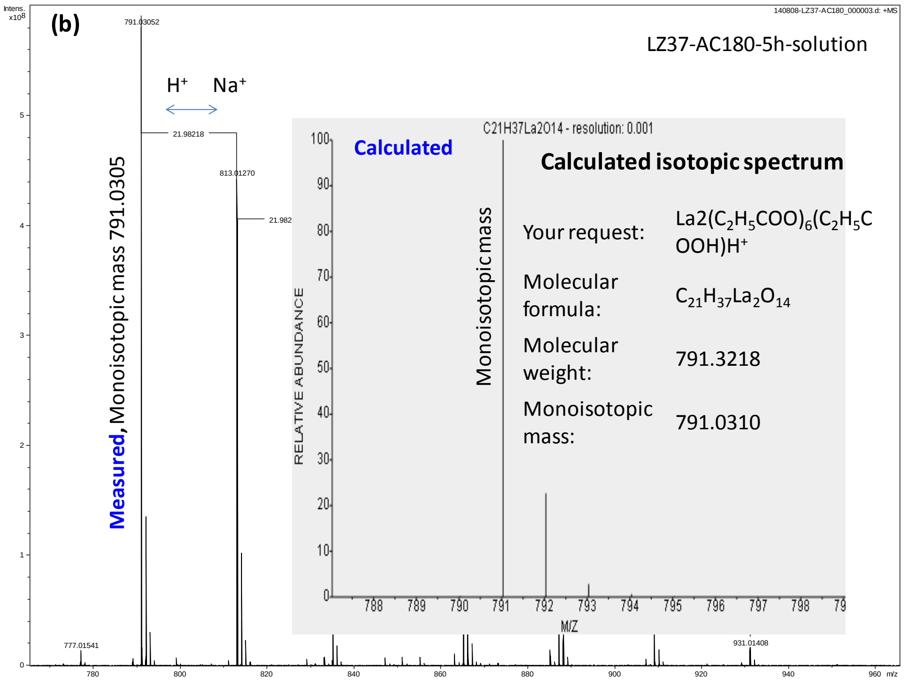
(b)
LZ37-AC180-5h-solution
140808-LZ37-AC180_000003.d: +MS
Intens. x10^8
791.03052
H+ Na+
21.98218
813.01270
21.982
777.01541
931.01408
Measured, Monoisotopic mass 791.0305
Calculated
C21H37La2O14 - resolution: 0.001
RELATIVE ABUNDANCE
Monoisotopic mass
M/Z
Calculated isotopic spectrum
Your request: La2(C2H5COO)6(C2H5COOH)H+
Molecular formula: C21H37La2O14
Molecular weight: 791.3218
Monoisotopic mass: 791.0310
m/z

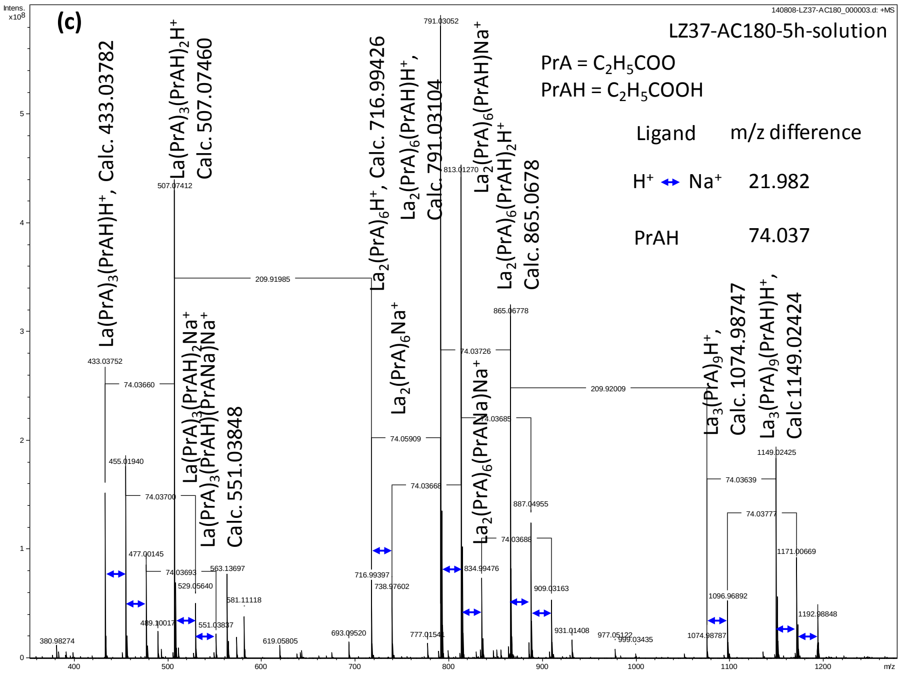
(c)
LZ37-AC180-5h-solution
140808-LZ37-AC180_000003.d: +MS
Intens. x10^8
PrA = C2H5COO
PrAH = C2H5COOH
Ligand m/z difference
H+ ↔ Na+ 21.982
PrAH 74.037
La(PrA)3(PrAH)H+, Calc. 433.03782
La(PrA)3(PrAH)2H+ Calc. 507.07460
La(PrA)3(PrAH)2Na+
La(PrA)3(PrAH)(PrANa)Na+ Calc. 551.03848
La2(PrA)6H+, Calc. 716.99426
La2(PrA)6(PrAH)H+, Calc. 791.03104
La2(PrA)6Na+
La2(PrA)6(PrAH)Na+
La2(PrA)6(PrANa)Na+
La2(PrA)6(PrAH)2H+ Calc. 865.0678
La3(PrA)9H+, Calc. 1074.98747
La3(PrA)9(PrAH)H+, Calc 1149.02424
433.03752
455.01940
477.00145
507.07412
529.05640
551.03837
563.13697
581.11118
619.05805
693.09520
716.99397
738.97602
777.01541
791.03052
813.01270
834.99476
865.06778
887.04955
909.03163
931.01408
977.05122
999.03435
1074.98787
1096.96892
1149.02425
1171.00669
1192.98848
380.98274
209.91985
209.92009
74.03660
74.03700
74.03693
74.05909
74.03666
74.03726
74.03685
74.03639
74.03777
m/z

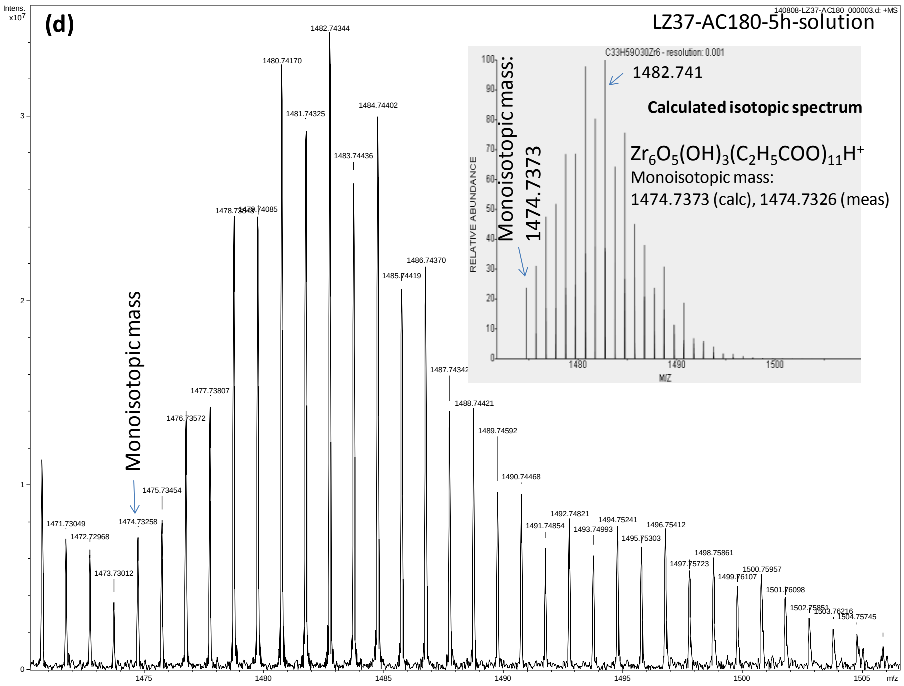
(d)
LZ37-AC180-5h-solution
140808-LZ37-AC180_000003.d: +MS
Intens. x10^7
Monoisotopic mass
1482.74344
1480.74170
1481.74325
1484.74402
1483.74436
1478.73878
1479.74085
1486.74370
1485.74419
1477.73807
1476.73572
1487.74342
1488.74421
1489.74592
1490.74468
1475.73454
1474.73258
1471.73049
1472.72968
1473.73012
1491.74854
1492.74821
1493.74993
1494.75241
1495.75303
1496.75412
1497.75723
1498.75861
1499.76107
1500.75957
1501.76098
1502.75851
1503.76216
1504.75745
m/z
C33H59O30Zr6 - resolution: 0.001
1482.741
Calculated isotopic spectrum
Monoisotopic mass: 1474.7373
RELATIVE ABUNDANCE
M/Z
$Zr_6O_5(OH)_3(C_2H_5COO)_{11}H^+$
Monoisotopic mass:
1474.7373 (calc), 1474.7326 (meas)

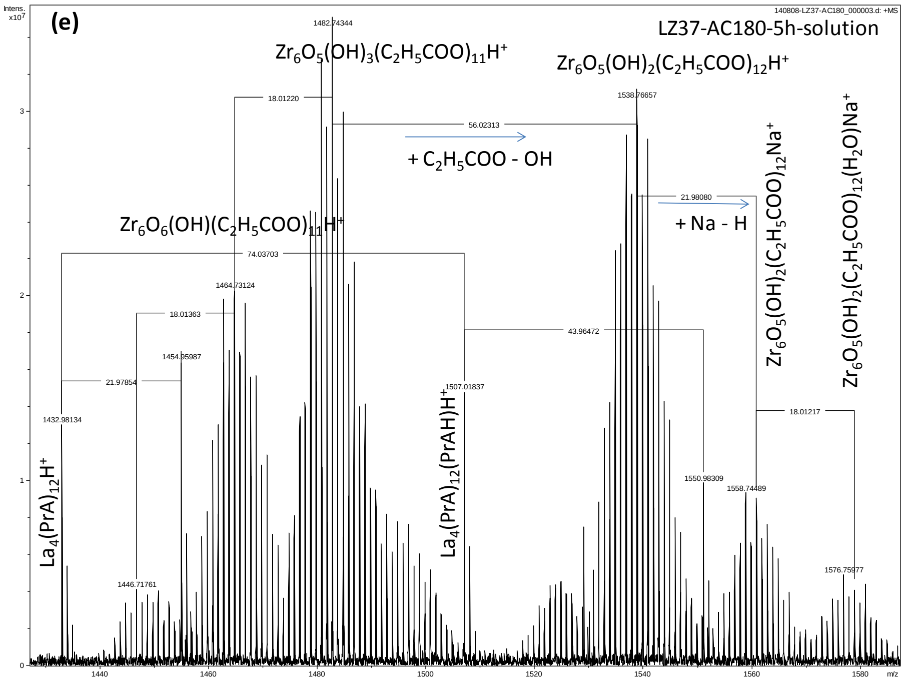
(e)
LZ37-AC180-5h-solution
140808-LZ37-AC180_000003.d: +MS
Intens. x10^7
1482.74344
$Zr_6O_5(OH)_3(C_2H_5COO)_{11}H^+$
$Zr_6O_5(OH)_2(C_2H_5COO)_{12}H^+$
1538.76657
18.01220
56.02313
$+ C_2H_5COO - OH$
21.98080
$+ Na - H$
$Zr_6O_5(OH)_2(C_2H_5COO)_{12}Na^+$
$Zr_6O_5(OH)_2(C_2H_5COO)_{12}(H_2O)Na^+$
$Zr_6O_6(OH)(C_2H_5COO)_{11}H^+$
74.03703
1464.73124
18.01363
1454.95987
21.97854
43.96472
1507.01837
$La_4(PrA)_{12}(PrAH)H^+$
1432.98134
$La_4(PrA)_{12}H^+$
18.01217
1550.98309
1558.74489
1446.71761
1576.75977
m/z

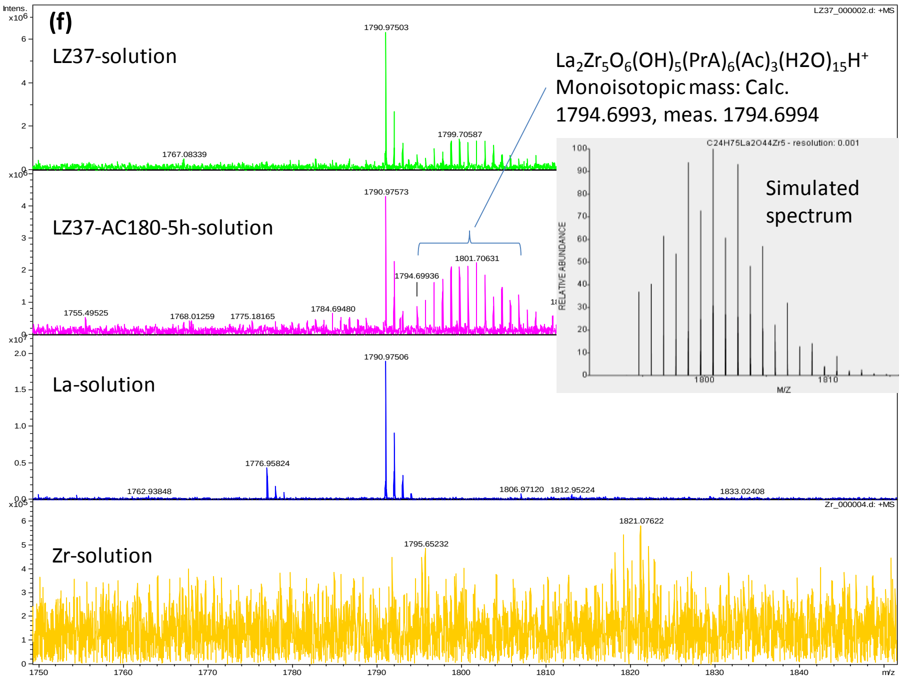

Figure S4. Details of the assignment of the MS spectra of (a–c) the La structures, (d–e) Zr structures, and (f) the LaZr structure.

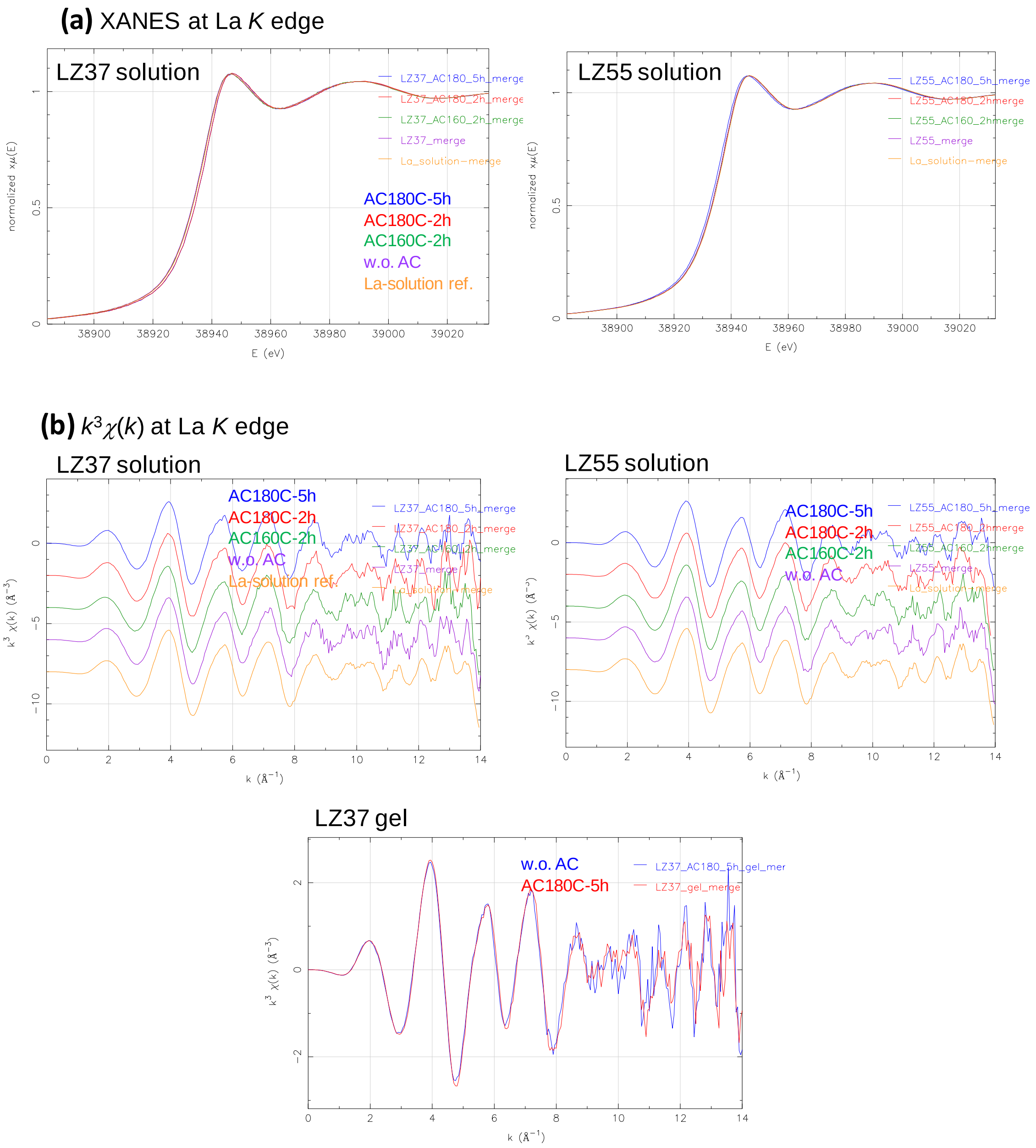
(a) XANES at La K edge
LZ37 solution
LZ37_AC180_5h_merge
LZ37_AC180_2h_merge
LZ37_AC160_2h_merge
LZ37_merge
La_solution−merge
AC180C-5h
AC180C-2h
AC160C-2h
w.o. AC
La-solution ref.
normalized xμ(E)
E (eV)
LZ55 solution
LZ55_AC180_5h_merge
LZ55_AC180_2hmerge
LZ55_AC160_2hmerge
LZ55_merge
La_solution−merge
(b) k³χ(k) at La K edge
LZ37 solution
LZ55 solution
k³ χ(k) (Å⁻³)
k (Å⁻¹)
LZ37 gel
w.o. AC
AC180C-5h
LZ37_gel_merge

(c) FT-XAFS at La *K* edge

LZ37 solution

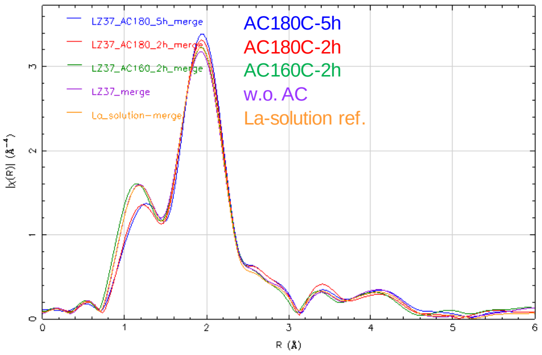

LZ55 solution

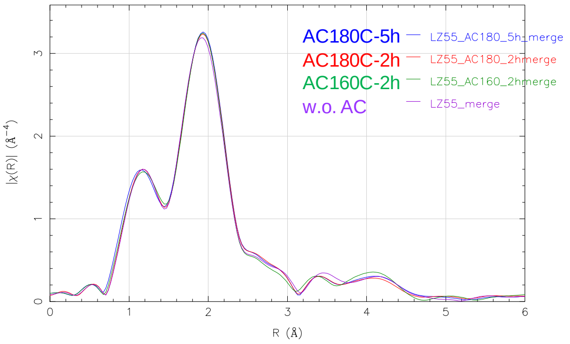

LZ37 gel

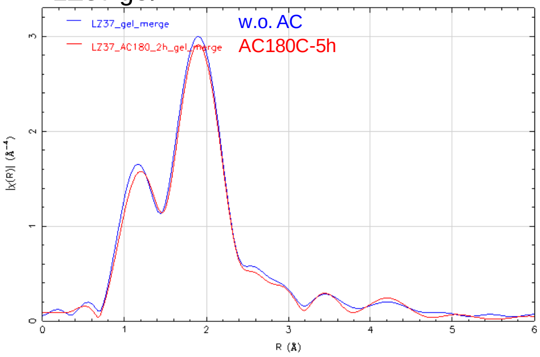

(d) XANES of powders at Zr *K* edge

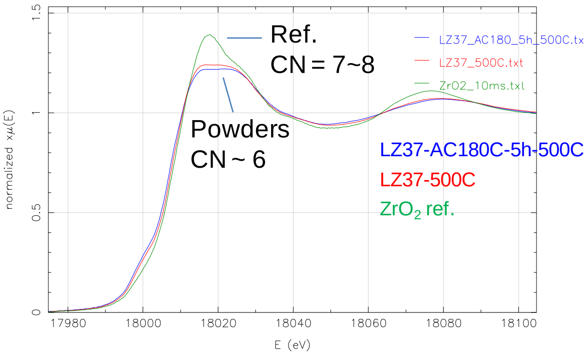

(e) $k^3\chi(k)$ at Zr K edge

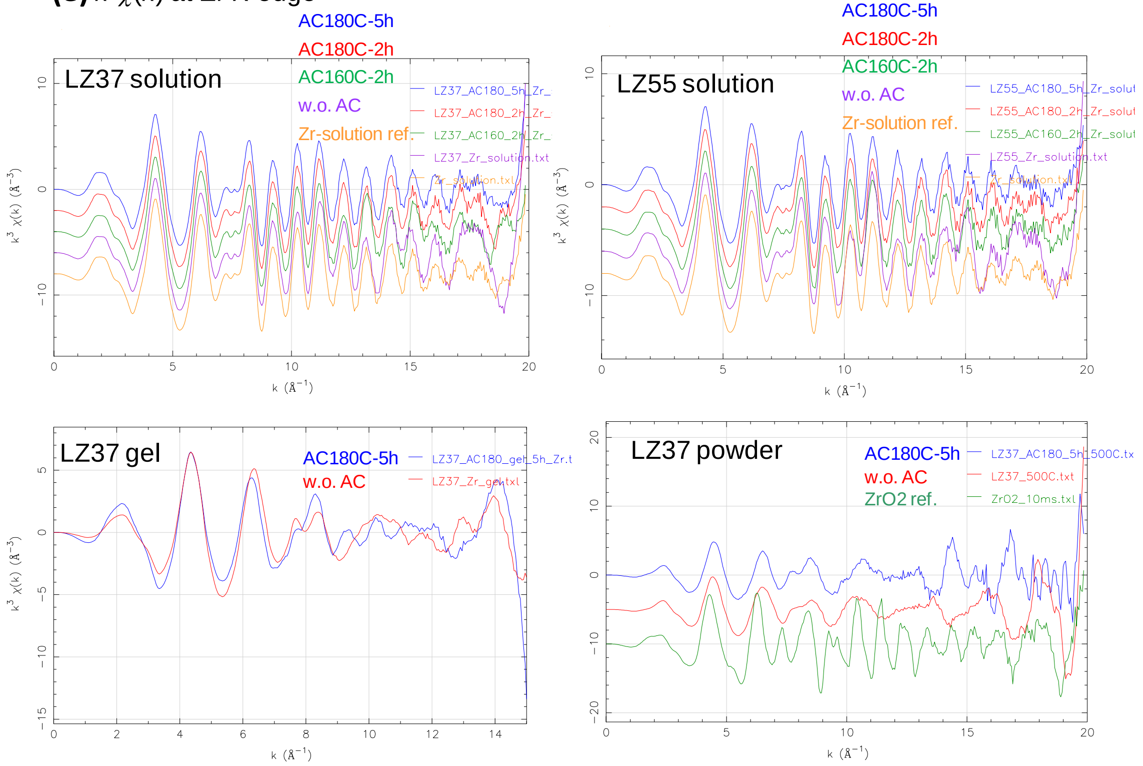

Figure S5. XAFS analysis. (a) XANES spectra of solutions at the La K edge. (b) $k^3\chi(k)$ spectra of solutions and gels at the La K edge. (c) FT-XAFS spectra of solutions and gels at the La K edge. (d) Comparison of the XANES spectra of powders at the Zr K edge with the reference monoclinic ZrO_2. (e) $k^3\chi(k)$ spectra at the Zr K edge.

References

S1. Momma, K.; Izumi, F. VESTA 3 for Three-Dimensional Visualization of Crystal, Volumetric and Morphology Data. *J. Appl. Crystallogr.* **2011***, 44*, 1272-1276.

S2. Puchberger, M.; Kogler, F.; Jupa, M.; Gross, S.; Fric, H.; Kickelbick, G.; Schubert, U., Can the Clusters $Zr_6O_4(OH)_4(OOCR)_{12}$ and $[Zr_6O_4(OH)_4(OOCR)_{12}]_2$ Be Converted into Each Other? *Europ. J. Inorg. Chem.* **2006**, 3283-3293.

S3. Martin, U.; Boysen, H.; Frey, F. Neutron Powder Investigation of Tetragonal and Cubic Stabilized Zirconia, TZP and CSZ, at Temperatures up to 1400 K. *Acta Crystallographica Section B* **1993**, *49*, 403-413
